# Systemic CLIP-seq analysis and game theory approach to model microRNA mode of binding

**DOI:** 10.1093/nar/gkab198

**Published:** 2021-04-06

**Authors:** Fabrizio Serra, Silvia Bottini, David Pratella, Maria G Stathopoulou, Wanda Sebille, Loubna El-Hami, Emanuela Repetto, Claire Mauduit, Mohamed Benahmed, Valerie Grandjean, Michele Trabucchi

**Affiliations:** Inserm U1065, C3M, Team Control of Gene Expression (10), Nice, France; Université Côte d’Azur, Inserm, C3M, Nice, France; Inserm U1065, C3M, Team Control of Gene Expression (10), Nice, France; Université Côte d’Azur, Inserm, C3M, Nice, France; Inserm U1065, C3M, Team Control of Gene Expression (10), Nice, France; Université Côte d’Azur, Inserm, C3M, Nice, France; Inserm U1065, C3M, Team Control of Gene Expression (10), Nice, France; Université Côte d’Azur, Inserm, C3M, Nice, France; Inserm U1065, C3M, Team Control of Gene Expression (10), Nice, France; Université Côte d’Azur, Inserm, C3M, Nice, France; Inserm U1065, C3M, Team Control of Gene Expression (10), Nice, France; Université Côte d’Azur, Inserm, C3M, Nice, France; Inserm U1065, C3M, Team Control of Gene Expression (10), Nice, France; Université Côte d’Azur, Inserm, C3M, Nice, France; Inserm U1065, C3M, Team Control of Gene Expression (10), Nice, France; Université Côte d’Azur, Inserm, C3M, Nice, France; Inserm U1065, C3M, Team Control of Gene Expression (10), Nice, France; Université Côte d’Azur, Inserm, C3M, Nice, France; Inserm U1065, C3M, Team Control of Gene Expression (10), Nice, France; Université Côte d’Azur, Inserm, C3M, Nice, France; Inserm U1065, C3M, Team Control of Gene Expression (10), Nice, France; Université Côte d’Azur, Inserm, C3M, Nice, France

## Abstract

microRNAs (miRNAs) associate with Ago proteins to post-transcriptionally silence gene expression by targeting mRNAs. To characterize the modes of miRNA-binding, we developed a novel computational framework, called optiCLIP, which considers the reproducibility of the identified peaks among replicates based on the peak overlap. We identified 98 999 binding sites for mouse and human miRNAs, from eleven Ago2 CLIP-seq datasets. Clustering the binding preferences, we found heterogeneity of the mode of binding for different miRNAs. Finally, we set up a quantitative model, named miRgame, based on an adaptation of the game theory. We have developed a new algorithm to translate the miRgame into a score that corresponds to a miRNA degree of occupancy for each Ago2 peak. The degree of occupancy summarizes the number of miRNA-binding sites and miRNAs targeting each binding site, and binding energy of each miRNA::RNA heteroduplex in each peak. Ago peaks were stratified accordingly to the degree of occupancy. Target repression correlates with higher score of degree of occupancy and number of miRNA-binding sites within each Ago peak. We validated the biological performance of our new method on miR-155-5p. In conclusion, our data demonstrate that miRNA-binding sites within each Ago2 CLIP-seq peak synergistically interplay to enhance target repression.

## INTRODUCTION

microRNAs (miRNAs) are transcribed as long RNAs and processed by Drosha and Dicer complexes into about 22 nucleotides (nt) long small RNA species, which are recruited by Argonaute proteins (Ago), including Ago2, to form the so-called miRNA-induced silencing complex (miRISC) (1). miRISC post-transcriptionally silences target RNAs by sequence pairing (1). In particular, miRNAs bind target RNA using either 6–8 nt in their 5′ end from the second nt (called seed sequence) (2,3) or portions of miRNA sequence outside the seed (4–6). The pairing miRNA sequence can perfectly match to the target sequence or can contain mismatches or bulges (2,3). Recently, it has been suggested that pairing of both 5′ and 3′ sequences of miRNA is prevalent in the majority of target sites and contributes to the specificity of the targeting (7). Importantly, a miRNA can potentially interact with many target-RNAs. At the same time, the complexity of the miRNA-dependent gene expression control is underscored by the fact that several miRNAs can potentially target one single target RNA, resulting in additive or synergistic effect. Considering that 2654 and 1978 miRNAs exist in human and mouse genomes, respectively (8), it appears that miRNAs and RNAs interact in multiple combinatorial manners to precisely control gene expression programs. Thus, a comprehensive identification of all miRNA-binding sites and their interplay is needed to fully understand the overall function of miRNAs in cells or tissues.

Cross-Linking ImmunoPrecipitation associated with high-throughput sequencing (CLIP-seq) is a recent technique to identify the direct binding sites of RNA-binding proteins in cells or tissues. When CLIP-seq is performed on Ago proteins enables researchers to characterize in a transcriptome-wide fashion the miRNA-binding sites with high resolution (5,9–11). The public availability of the Ago CLIP-seq data coupled with the RNA profiling allows systemic assessment of the combinatorial mechanism for multiple miRNA targeting associated with transcriptomic silencing. This task would be beneficial to improve the computational prediction of miRNA-binding sites and to derive precious biological information about the global function of miRNAs in a cell or tissue-specific fashion.

In this study, we present a quantitative method that integrates multiple miRNA-binding activity and gene expression data and propose a model for miRNA mode of action.

To identify miRNA-binding sites for endogenously expressed miRNAs, we analyzed eleven publicly available Ago2 CLIP-seq datasets from raw data using a computational framework called optiCLIP (optimized pipeline of CLIP-seq data analysis) based on a novel benchmarked workflow for pre-processing and peak calling (12), peaks reproducibility assessment among replicates by a newly developed strategy based on Jaccard index calculation, and finally employing miRBShunter prediction program for miRNA-binding sites identification (4).

To evaluate the performance of optiCLIP, we repeated the analysis using other prediction programs, namely, TargetScan (13), miRanda (14), TarPmiR (15) and RNAhybrid (16).

We have also set up a quantitative model to study the interplay of miRNA-binding sites, by proposing a degree of occupancy that stratifies Ago2 peaks. To this goal, we have developed a new algorithm, called miRgame, to quantify the degree of occupancy by taking into account the number of miRNA-binding sites, number of miRNAs targeting each binding site (promiscuous miRNA-binding sites), and the free energy of each miRNA::RNA heteroduplex within each Ago2 peak.

The final aim of the present work is to demonstrate the ability of optiCLIP framework and miRgame quantitative model to provide a more accurate interpretation of miRNA-dependent gene expression control and decipher its complexity.

## MATERIALS AND METHODS

### optiCLIP: an integrated and optimized framework for Ago2 CLIP-seq data analysis

In order to analyze Ago2 CLIP-seq experiments from raw data files and identify miRNA-binding sites, a computational framework, called optiCLIP, was developed thanks to our recent computational benchmarks of several software used in CLIP-seq analysis workflow (4,12). optiCLIP starts with raw data preprocessing that includes adapter removal with cutadapt (17), quality filters of reads to identify possible sequencing errors or biases requiring a minimal quality score of 15 and a minimal length of 10 nucleotides, and duplicate collapsing with Prinseq (18) with the option ‘-derep 123’ to collapse duplicated reads, ‘-min_qual_mean 15 –min_len 10’ to finally select reads based on quality and length. The alignment of sequence reads to a reference genome is done using Novoalign software (http://www.novocraft.com/products/novoalign/) with specific parameters set up for Ago2 CLIP-seq (12,19):‘-t 85’ the alignment cost that allow two substitutions, two consecutives deletions or one substitution in addition to one deletion, ‘-l 15’ requires more than 15 high-quality matches, ‘-s 1’ is the computation step, and ‘-o sam’ output format. Finally, peaks have been identified by Pyicoclip (20) as the best performing software for Ago2 CLIP-seq peak calling (4).

To identify high-confidence and reproducible peaks, we have developed a novel strategy that considers experimental replicates. Briefly, instead of merging together all the reads coming from different replicates before performing the peak calling (for simplicity, here called the merge method), as it has been done to date by the community (21–23), we reasoned that a good strategy to find high-confidence peaks could be to perform the peak calling step in each replicate separately and then quantify the overlap of peaks among multiple replicates through the Jaccard index (*J*-index) calculation. The boundaries of the consensus peaks are then calculated by the union of all the peaks that fulfill the reproducibility constrains. To assess the optimal *J*-index threshold, we tested three different *J*-index thresholds, namely, the 20%, 40% and 80% (here called *J*20, *J*40 and *J*80, respectively). As last step, optiCLIP identifies the miRNA-binding sites from the identified peaks, using miRBShunter that identifies all potential miRNA::RNA heteroduplexes for both seed and non-seed mode of binding.

Heuristic approach was used to adapt the parameters of miRBShunter to datasets with different sizes:

- <100 peaks: -log_10_(***p***) = 3; ***ms***= 0.25; ***Fimo_score***= 0.0008
- 100 < peaks < 1000: −log_10_(***p***) = 5; ***ms***= 0.35; ***Fimo_score***= 0.0008
- 1000 < peaks < 2000: −log_10_(***p***) = 7; ***ms***= 0.35; ***Fimo_score***= 0.0002
- >2000 peaks: -log_10_(***p***) = 12; **ms**= 0.35; ***Fimo_score***= 0.0002

where ***p*** is the *P*-value calculated by Homer (24), ***ms*** is the match score calculated by Homer between the identified motifs and the miRNA sequences, the ***Fimo_score*** is the threshold for the program FIMO of the MEME suite (25) to localize the motifs in the peak.

OptiCLIP is written mainly in python language and is freely available at https://github.com/TrabucchiLab/optiCLIP.

To assess the performance of the miRNA prediction step of optiCLIP, we repeated the analysis using other prediction programs, namely, TargetScan, miRanda, TarPmiR and RNAhybrid, using the default parameters.

### Specificity in identifying miRNA-binding sites on Ago2 peaks

To evaluate the specificity of the identified miRNA-binding sites, we have randomized five times the sequence of the 25 most expressed miRNAs of each dataset by using the Shuffle DNA software (www.bioinformatics.org/sms2/shuffle_dna.html), thus five creating negative control datasets, and used with peaks identified by *J*20 threshold and the merge method to feed the prediction programs. The number of identified miRNA-binding sites using miRBShunter, miRanda and TargetScan by the real sequence of miRNAs (reference dataset) was then compared with each of the negative control datasets (named ‘shuffles’) Wilcoxon signed-rank test and boxplots were generated by R software.

### Precision of miRNA-binding sites

The precision of the prediction can be measured by the distance from the miRNA-binding sites and the center of the peaks. The precision has been calculated for miRBShunter, TargetScan and miRanda for all datasets on peaks identified by *J*20 threshold or merge methods. miRNA-binding sites close to the peak center correspond to high degree of precision (4,26). The comparison between the three prediction programs, namely, miRBShunter, miRanda, and TargetScan, was performed by using the Kruskal-Wallis test. Boxplots were generated by R software.

### Publicly available Ago2 HITS-CLIP datasets

Raw data for Ago2 CLIP-seq datasets were downloaded from GEO database (Table 1) with the exception of Chi *et al.* (5) datasets, which were downloaded from http://ago.rockefeller.edu/rawdata.php

**Table 1.** Ago2 CLIP-seq datasets

GEO accession	Cell lines/tissue	Species	Replicates number	Reference
GSE28865	HEK293	Human	2	(48)
GSE42701	HeLa	Human	2	(49)
GSE52084	Brain samples, cingular gyrus	Human	6	(50)
GSE52084	Brain samples, motor cortex	Human	5	(50)
GSE83410	Heart samples, ventricle	Human	6	(51)
GSE44404	293S	Human	4	(52)
GSE41285	CD4 + T-cells	Mouse	12	(35)
GSE85219	P19 mESC + mmu-let-7a-5p	Mouse	3	(36)
GSE25310	mESC	Mouse	3	(53)
http://ago.rockefeller.edu	Brain samples, cortex	Mouse	3	(5)
http://ago.rockefeller.edu	Brain samples, cortex	Mouse	2	(5)

### Alignment and quantification of the most expressed miRNAs

To identify the most expressed miRNAs in the eleven datasets, we selected reads from 18 to 35 nt in length and mapped the reads using Novoalign software with highly stringent parameters:

novoalign -c 20 -t 20 -l 5 -s 1 -F STDFQ -o sam -r All -d INDEX_GENOME -f input_file > output_file.

Then, we quantified the number of mapped reads for each miRNA, reads count were normalized for each replicate using the quantile normalization. We finally calculated the mean among replicates and ranked them to obtain the 25 most expressed miRNAs for each dataset (Supplemental Table S1).

### Clustering the miRNA-binding preferences for each miRNA

Clustering analysis was performed in order to investigate the miRNA mode of binding. miRNA sequence positions that matched to target sequence were plotted as heatmap of the frequency. Heatmaps and dendrograms were generated by R software using the functions ‘hclust’ or ‘K-means’ with ‘euclidean’ distance metrics and ‘complete’ method.

### Expression data analysis

Raw data from microarray gene expression profiles were downloaded from GEO database or specific websites (Table 2) and analyzed using the packages ‘limma’ (27) and ‘affy’ (28) from Bioconductor and R software.

**Table 2.** Microarray datasets

GEO accession	Cell line	Species	Replicates number	miRNA	Reference
GSE41285	CD4+ T-cells	Mouse	1	155-5p	(35)
GSE89033	P19 cells	Mouse	3	Let-7a-5p	(36)
http://psilac.mdc-berlin.de	HeLa cells	Human	3 for each miRNA	1-3p; 16-5p	(37)
GSM302945	HeLa cells	Human	1	124-5p	(5)

### miRgame: degree of occupancy for Ago2 CLIP-seq peaks

The goal of the degree of occupancy is to provide a stratification of the Ago2 peaks, which silencing of the target mRNAs would be positively correlated. To establish a quantitative model to calculate the degree of occupancy of Ago2 peaks, we made two assumptions:

- the identified miRNA-binding sites do not depend on the miRNA expression levels since we identified the binding sites only of the most expressed miRNAs in each dataset (up to the top 25 mostly expressed miRNAs for each dataset);
- miRNA-binding sites within the same peak synergistically cooperate.

The first assumption implies that we can ignore the concentration levels of each miRNA considered in the analysis. The second assumption implies we can disregard the distance among miRNA-binding sites within the same Ago2 peak. Although the distance plays a role in terms of cooperation between miRNA-binding sites considering a range distance of 8–60 nucleotides (29,30,41), we have made this assumption based on the fact that the majority of miRNA-binding sites do not overlap to each other and show overall median distances of not overlapping sites within a synergistic cooperation. Thus, because they can potentially cooperate to synergistically enhance target repression. We termed an Ago2 peak as a ‘miRNA-binding unit’, for which the degree of occupancy is calculated.

To model the degree of occupancy of each Ago2 peak, we summarized in one single value the contribution of each miRNA-binding site and the energy of each miRNA::RNA heteroduplex. We applied a game theory approach to provide a value of the degree of occupancy, considering that each miRNA-binding site is a ‘player’ of the game and all miRNAs that bind the same binding site are the different ‘coalitions’. In this ‘game’ the weight of the marginal contribution of each player (the miRNA-binding site) is modulated by the number of the possible coalitions that each player can make (the number of miRNAs for the same binding site) and by the miRNA-binding energy of each possible miRNA::RNA heteroduplex. This game (quantitative model) is defined by the following equation for predictions using miRBShunter:

$$\begin{equation*}{\Delta _\pi }\ \left( {m,n,E_i^\nu ,nt_i^\nu } \right) = \mathop \sum \limits_\nu {\mu _\nu }\ \left( {n,E_i^\nu ,nt_i^\nu } \right)\end{equation*}$$

where $\pi$ is the peak, ${\Delta _\pi }$ is the degree of occupancy, $m$ is the number of miRNA-binding sites in the considered peak, $n$ is the number of miRNAs that bind the same binding site, ${E_i}$ is the minimum free energy of the miRNA::RNA heteroduplex calculated by miRBShunter, and $n{t_i}$ represents the characteristic of the duplex structure (namely, number of nucleotides paired, presence of mismatches and/or bulges). To calculate the marginal contribution of each coalition for each miRNA-binding site, we defined the following function:

$$\begin{equation*}{\mu _\nu }\ \left( {n,{E_i},n{t_i}} \right) = \log\left( {\mathop \sum \limits_i \beta \left( {{E_i},n{t_i}} \right)} \right)\end{equation*}$$

where $\beta ( {{E_i},n{t_i}} )$ = (−MFE/Min(MFE)) + *N*_paired_nt/len_miRNA_ + *N*_paired_nt_seed/len_seed_ + *N*_paired_nt_motif/ len_motif_ + (len_seed_ − *N*_bulges_seed)/len_seed_ is the miRNA::RNA heteroduplex by miRBShunter. This score is based on the following parameters (4):

1. MFE is the free energy of the most stable structure given by RNAduplex tool (31);
2. *N*_paired_nt is the number of paired nucleotides in the predicted heteroduplex;
3. *N*_paired_nt_motif is the number of paired nucleotides in the motif found with Homer software;
4. *N*_paired_nt_seed is the number of paired nucleotides in the seed region;
5. *N*_bulges_seed is the number of bulges in the heteroduplex in the seed;
6. len_miRNA_ is the length of the miRNA sequence;
7. len_seed_ is the length of the miRNA seed;
8. len_motif_ is the length of the motif found with Homer software.

This algorithm is an adaptation of the game theory approach (32,33).

miRgame was also adapted for miRanda and TargetScan to compare the data obtained with these prediction tools and miRBShunter data. The marginal contribution score of each coalition for each miRNA-binding site was considered as equal to the relative score calculated by miRanda and the context score for TargetScan, respectively.

The use of the logarithmic scale to sum the contribution of each miRNA::RNA heteroduplex within the same binding site would lower down the *μ_ν_* score of binding sites targeted by many miRNAs, reasoning that these binding sites would be less regulated by expression changes of single targeting miRNAs, but by the pool of targeting miRNAs, as it has been previously observed (34). To validate the miRNA degree of occupancy, we used the gene expression profiles whereby single miRNAs were either knocked out or overexpressed.

The model miRgame was run on the *J*20 threshold and merge method peaks and the related binding sites predicted by miRBShunter, miRanda or TargetScan for the considered datasets.

To investigate how the degree of occupancy correlates to target repression, we determined four levels of degree of occupancy for each prediction program based on quartiles for 3′UTR peaks.

We then investigated how the four levels correlated to target repression using the expression data (Table 2) available for mmu-miR-155-5p.

To test the repression of the target mRNAs in each level compared to the cumulative distribution of all genes (background) we used the Kolmogorov–Smirnov test.

The miRgame analyses were performed in R software. miRgame scrips are shown in the Supplemental data 1.

### Program implementation

All software were installed and run on linux workstation with two 2.6 GHz Intel Xeon Ubuntu machine equipped with 4 × 32GB of RAM.

## RESULTS

### Identification of miRNA-binding sites by *J*-index method and its sensitivity

We collected and analyzed eleven Ago2 CLIP-seq datasets (Table 1) generated from human or mouse cells or tissues and analyzed to identify miRNA-binding sites. In Supplemental Figure S1 is illustrated the computational framework we used for this analysis and named it optiCLIP (optimized pipeline of CLIP-seq data analysis), which is freely downloadable at https://github.com/TrabucchiLab/optiCLIP (see Materials and Methods for details).

**Figure 1.**
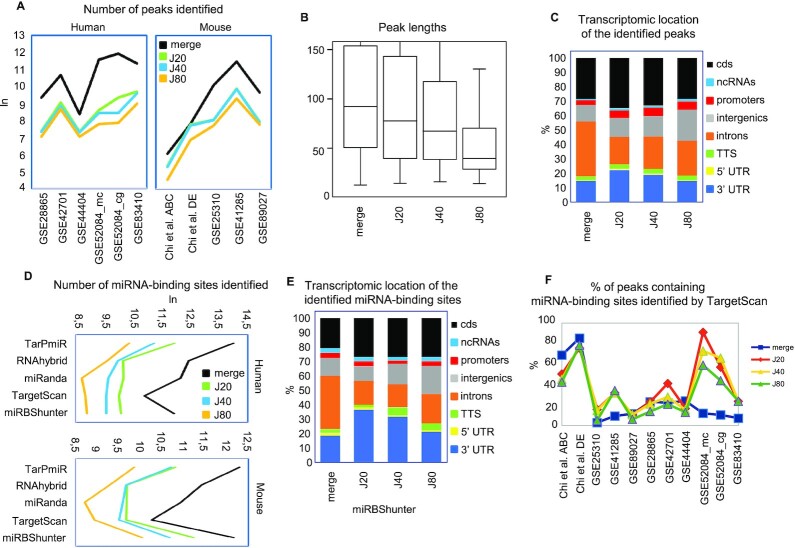
Evaluation of Jaccard-index thresholds to identify reproducible peaks and comparison with the merge method. (**A**) Number of peaks, (**B**) peak lengths, and (**C**) transcriptomic distributions of Ago2 peaks identified by the merge method and the indicated Jaccard-index (*J*) thresholds. (**D**) Sensitivity and (**E**) transcriptomic distributions of the miRNA-binding sites identified with the indicated prediction programs. (**F**) Line graph showing the percentage of peaks containing miRNA-binding sites identified by the high-confidence TargetScan program on peaks identified by the indicated J thresholds and the merge method for the indicated Ago2 CLIP-seq datasets.

We tested three different J-index thresholds, namely, the 20%, 40% and 80% (here called *J*20, *J*40 and *J*80, respectively), by quantifying the number and length of the identified peaks (Figure 1A and B). In human datasets, the *J*20 threshold identified a significantly higher number of peaks in human datasets compared to *J*40 and *J*80 thresholds (*P*< 0.05, Wilcoxon signed-rank test). In mouse datasets, the number of peaks identified by *J*20 threshold was higher compared to *J*40 and *J*80 thresholds but not significant (*P* = 0.062, Wilcoxon signed-rank test) (Figure 1A). Furthermore, the peaks identified by the *J*20 threshold had a significantly wider length compared to the two other *J*-index thresholds (*P*< 0.001, Wilcoxon rank sum test) (Figure 1B). As expected, the number of identified peaks by merge method was higher and the length wider compared to the *J*20 because less stringent (Figure 1A, B, and Supplemental data 2).

Considering the transcriptomic locations of the identified peaks, we observed a significant enrichment of the 3′UTR location in *J*20 threshold compared to *J*40 and *J*80 thresholds and the merge method (*P*< 0.01, Wilcoxon signed-rank test) (Figure 1C).

Next, we detected miRNA-binding sites using miRBShunter and other four prediction programs namely, TargetScan, miRanda, RNAhybrid, and TarPmiR (Supplemental data 2). As shown in Figure 1D, the merge method outperformed the *J*-index method in number of binding sites, while *J*20 performed better than the other two *J*-index thresholds for all prediction programs in human datasets (*P*< 0.05, Wilcoxon signed-rank test). Similar results were observed in mouse datasets although the differences among the methods were not significant (*P* = 0.062, Wilcoxon signed-rank test).

Then, we inspected the transcriptomic location of the identified miRNA-binding sites. As shown in Figure 1E and Supplemental Figure S2A, B and C, miRNA-binding sites were enriched in the 3′UTR in *J*20 threshold compared to *J*40 and *J*80 (Supplemental Table S2), and the merge method for all prediction programs.

In order to compare the different methods, we calculated the percentage of peaks containing high confidence TargetScan predicted miRNA-binding sites. The percentage of peaks containing TargetScan miRNA-binding sites was overall significantly higher in *J*20 compared to *J*80 threshold (*P* = 0.013, Wilcoxon signed-rank test) (Figure 1F). Similarly, the percentage of peaks containing miRNA-binding sites identified by miRBShunter (Supplemental Figure S2D) and by miRanda (Supplemental Figure S2E) was overall significantly higher in *J*20 compared to *J*40 and *J*80 thresholds (*P*< 0.05, Wilcoxon signed-rank test).

Together, these analyses demonstrate that, among the different *J*-index thresholds, the *J*20 outperformed in terms of sensitivity and accuracy. Furthermore, although the merge method identified much more miRNA-binding sites, the *J*20 threshold looks more accurate to find a relative enrichment of miRNA-binding sites located in the 3′UTR. Because of these results, we decided to focus our analysis by comparing just the *J*20 threshold to the merge method. Moreover, in these analyses, miRBShunter, miRanda, and TargetScan performed much better compared to TarPmiR and RNAhybrid. Therefore, from this point of the manuscript, we also focus our investigation on miRBShunter, miRanda and TargetScan.

### Specificity and precision in identifying miRNA-binding sites on Ago2 peaks

**Figure 2.**
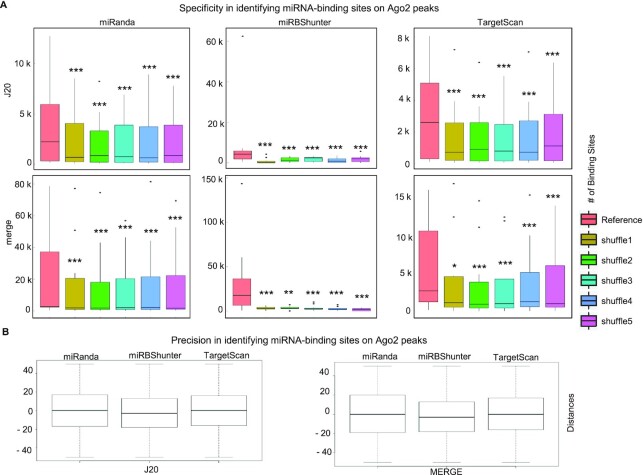
Specificity and precision in identifying miRNA-binding sites on Ago2 peaks identified by *J*20 or merge methods. (**A**) Specificity. Boxplot showing the number of miRNA-binding sites identified by miRanda, miRBShunter or TargetScan using the original miRNA sequence datasets (Reference) or five randomly permuted miRNA sequences (shuffles) datasets, as indicated. Wilcoxon signed-rank test was applied to compare reference versus each shuffle. *P* < 0.05 (*), *P* < 0.01 (**), *P* < 0.001 (***), not significant (ns). (**B**) Precision. Boxplot showing the distance between the miRNA-binding site center for the top expressed miRNAs and the peak center for the indicated prediction programs on either the *J*20 (left panel) or the merge (right panel) methods.

Concerning the specificity, we performed five sequence randomizations of the top expressed miRNAs in each dataset, identified the miRNA-binding sites in each randomized dataset, and compared them to those found using the original datasets. We used TargetScan, miRanda and miRBShunter prediction programs and both *J*20 threshold and merge methods. Importantly, we found that the number of miRNA-binding sites was significantly much higher in the original datasets compared to the randomized ones (Figure 2A), indicating that all the three prediction programs are specific.

Furthermore, as shown in Figure 2B, all three prediction programs have identified miRNA-binding sites that are close to the center of the related peaks using *J*20 threshold and merge methods. The difference between the prediction programs was not significant indicating they are comparable in terms of precision (Wilcoxon signed-rank test).

### Clustering the mode of binding of miRNAs

We clustered the data generated by the optiCLIP framework to investigate the miRNA mode of binding in a transcriptome-wide fashion. We plotted the binding sequence position for each miRNA as heatmap of the frequency using the ‘hclust’ function. Dendrograms were obtained using *K*-means function.

The clustering of miRNA-binding sites was applied to human and mouse datasets using miRBShunter, TargetScan, and miRanda prediction programs on peaks identified by *J*20 threshold and the merge method.

**Figure 3.**
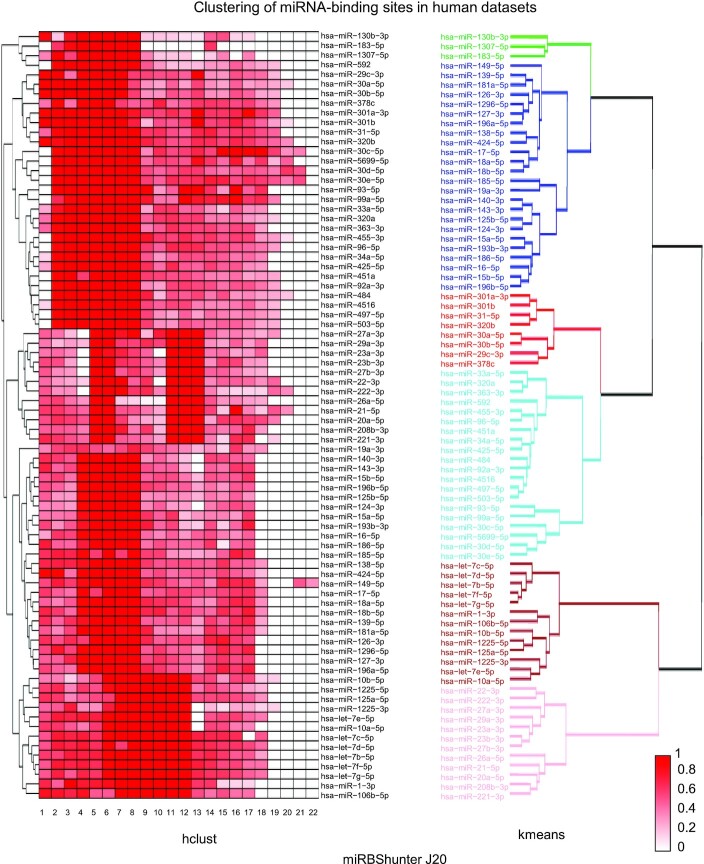
Clustering of miRNA-binding sites in human datasets. (left panel) Heatmap showing the frequency of the binding positions in the sequence of human miRNAs identified by miRBShunter using *J*20 threshold for peak identification. miRNAs are grouped by hierarchical clustering using ‘hclust’ by R software. The color intensity (scale range from 0 to 1) represents the normalized frequency for each miRNA. (right panel) *K*-means dendrogram with group colors showing the clusterization of human miRNA-binding sites identified by miRBShunter applied to the *J*20 threshold peaks.

As shown in Figure 3A, miRNA-binding sites identified by miRBShunter are focused on the seed sequence just for a subset of miRNAs considering the *J*20 threshold in human datasets, while in merge method the subset of seed-focused binding sites was smaller (Supplemental Figure S3A). These results indicate that the miRNA mode of binding predicted by miRBShunter show heterogeneity among different miRNAs.

As expected, for TargetScan and miRanda the weight of the miRNA seed in predicting binding sites was much higher respect to the rest of the miRNA sequence for both *J*20 threshold and the merge method (Supplemental Figures S4A−S7A). In mouse datasets, we observed a variety of mode of binding with no specific subgroup focused on the seed sequence for binding sites identified by miRBShunter in both *J*20 and merge method (Supplemental Figures S8A and S9A), while for TargetScan and miRanda seed binding was again the enriched mode of binding (Supplemental Figures S10A–S13A). Concerning the clustering method, we found similar clustering between hclust and *K*-means methods for the considered three prediction programs, in both *J*20 index threshold and merge method for human and mouse datasets (Figure 3, Supplemental Figures S3–S13).

Together, these data indicate that for miRBShunter the predicted miRNA mode of binding looks heterogenous while for TargetScan and miRanda all miRNAs mainly bind with the seed sequence. These results are independent on the clustering method used.

### Effectiveness of miRNA-dependent repression in the 3′UTR

Reasoning that the data we generated with optiCLIP might provide a base for the definition of novel types of miRNA-binding sites to be used for target prediction, we investigated the functionality of these sites in mediating target silencing. To carry out this analysis, we focused on the following miRNAs, mouse miR-155-5p (35) and let-7a-5p (36), and human miR-1-3p, miR-16-5p (37), and miR-124-3p (5), whose differential RNA profile data upon miRNA overexpression or knockout/down are available (Table 2).

**Figure 4.**
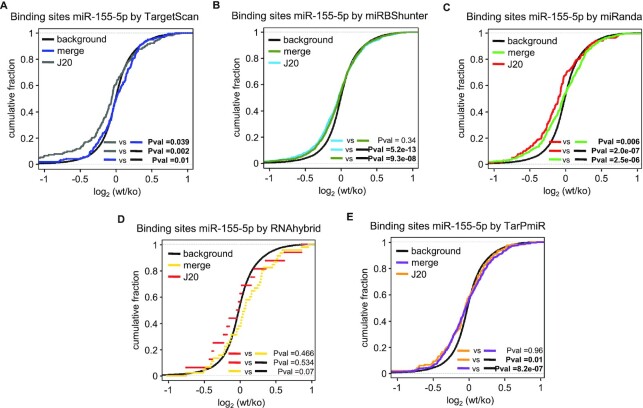
Specific miRNA mode of bindings in 3′UTR triggers target repression. Cumulative distributions showing target repression upon mmu-miR-155-5p knockout in T cells for TargetScan (**A**), miRBShunter (**B**), miRanda (**C**), RNAhybrid (**D**) and TarPmiR (**E**). The *P*-value was calculated using Kolmogorov−Smirnov statistical test for target downregulation comparing *J*20 threshold, merge method and background (cumulative distribution of all genes).

We investigated the repression efficacy of the mRNAs containing miRNA-binding sites in the 3′UTR exploiting the microarray expression data for miRBShunter and the other four prediction programs, namely, TargetScan, miRanda, RNAhybrid and TarPmiR. We studied the miRNA-dependent repression of target mRNAs checking the significance when comparing the *J*20 threshold with the merge method and both of them with the cumulative distribution of all genes (background/black line). As shown in Figure 4, for TargetScan and miRanda, we found that *J*20 threshold outperformed the merge method (*P* = 0.039 and *P* = 0.006, respectively, Kolmogorov–Smirnov test) (Figure 4A and C). Target repression between the *J*20 threshold and the merge method was not significant for miRBShunter, RNAhybrid and TarPmiR (*P* = 0.34, *P* = 0.46 and *P* = 0.96, respectively, Kolmogorov–Smirnov test) (Figure 4B, D and E). However, when we compared both methods with the background, we observed that all prediction programs showed significant results (Figure 4A, B, C and E), except for RNAhybrid (Figure 4D). In Supplemental Figures S14 and S15, we plotted the cumulative fractions of the other four miRNAs. Comparing *J*20 threshold with the merge method, we noticed significant values for miRBShunter considering mmu-let-7a-5p (*P* = 0.037, Kolmogorov–Smirnov test) and TargetScan, miRBShunter and RNAhybrid for hsa-miR-124-3p (*P* = 0.01, *P* = 0.0004 and *P* = 0.042, respectively, Kolmogorov–Smirnov test). Comparing either *J*20 threshold or the merge method with background, mmu-let-7a-5p target repression was significant for miRBShunter (*P* = 0.004 and *P* = 0.01, respectively, Kolmogorov–Smirnov test), while for TarPmiR and RNAhybrid the merge method outperformed (*P* = 0.03 and *P* = 0.03, respectively, Kolmogorov–Smirnov test) (Supplemental Figure S14A). For hsa-miR1-3p, only the merge method using miRanda program gave significant values (*P* = 0.025, Kolmogorov–Smirnov test) (Supplemental Figure S14B). Concerning hsa-miR16-5p, both *J*20 threshold and merge methods led to significant target repression results for TargetScan (*P* = 0.007 and *P* = 0.0003, respectively, Kolmogorov–Smirnov test) and miRanda (*P* = 0.017 and *P* = 4.3×10^–10^, respectively, Kolmogorov–Smirnov test), while for miRBShunter and TarPmiR merge method gave significant results (*P* = 0.002 and *P* = 0.06, respectively, Kolmogorov–Smirnov test) (Supplemental Figure S14C). Regarding hsa-miR-124-3p, significant values were obtained for TargetScan, miRBShunter and RNAhybrid when comparing J20 threshold with background (*P* = 0.0002, *P* = 0.0002 and *P* = 0.042, respectively, Kolmogorov–Smirnov test) (Supplemental Figure S15).

These results overall indicate that miRNA-binding sites identified by the *J*20 threshold are functional in terms of mediating target silencing. Although *J*20 threshold overall outperformed compared with the merge method, different experimental conditions (i.e. different antibody used for immunoprecipitation) or computational conditions prediction (i.e. different prediction programs) may impact on the results of the analysis.

In these analyses, once again, we noticed that miRBShunter, miRanda, and TargetScan performed much better compared to TarPmiR and RNAhybrid. Therefore, from this point of the manuscript, we decided to pursue our investigation on just miRBShunter, miRanda, and TargetScan.

Importantly, miRNA-binding sites identified in other transcriptomic regions did not bring any repression on target RNAs with any prediction programs (data not shown).

### Multiple and promiscuous miRNA-binding sites within Ago2 CLIP-seq peaks

To further investigate the miRNA-binding activity, we quantified the number of multiple miRNA-binding sites and the number of binding sites targeted by more than one miRNA (promiscuous binding sites) in each Ago2 peak.

**Figure 5.**
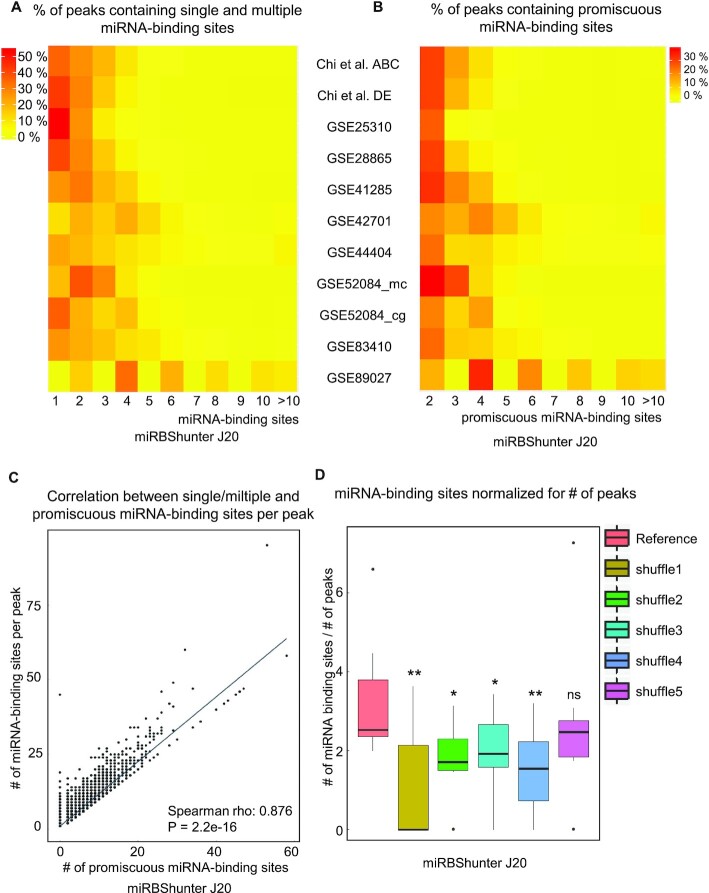
Multiple and promiscuous miRNA-binding sites in Ago2 CLIP-seq peaks. (**A**) Heatmaps showing the percentage of peaks containing single and multiple miRNA-binding sites or (**B**) containing promiscuous miRNA-binding sites identified by miRBShunter on the *J*20 threshold peaks for the indicated Ago2 CLIP-seq datasets. (**C**) Plot showing the significant correlation (Spearman rho: 0.876) between the single/multiple and the promiscuous miRNA-binding sites per peak identified by miRBShunter on the *J*20 threshold peaks. (**D**) Boxplot showing the number of miRNA-binding sites identified by miRBShunter on the *J*20 threshold peaks, normalized for the number of peaks on the indicated datasets. Wilcoxon signed-rank test was applied to compare reference with each shuffle. *P* < 0.05 (*), *P* < 0.01 (**), *P* < 0.001 (***), not significant (ns).

We observed that 46−100% and 46−84% of the peaks identified by *J*20 threshold and the merge method contain one or multiple miRBShunter-identified miRNA-binding sites, respectively (Figure 5A and Supplemental Figure S16A). Concerning miRanda, the related percentages were 22−56% and 24−56%, while for TargetScan the percentages were 3−32% and 3−32%, respectively (Supplemental Figures S17A, S18A, S19A and S20A).

In addition, we found that 32−100% and 27−84% of the peaks identified by *J*20 and the merge method contain promiscuous miRBShunter-identified miRNA-binding sites, respectively (Figure 5B and Supplemental Figure S16B). For miRanda these percentages were 5−31% and 6−33% and for TargetScan were 3−27% and 2−32%, respectively (Supplemental Figures S17B, S18B, S19B and S20B).

Next, we plotted the number of multiple miRNA-binding sites identified by miRBShunter, miRanda and TargetScan per peak as a function of the number of promiscuous miRNA-binding sites for *J*20 threshold and the merge method (Figure 5C and Supplemental Figures S16C, S17C, S18C, S19C and S20C). We found a linear dependency with Spearman correlation score between these two variables for all prediction programs and for both *J*20 threshold and the merge method. To validate these results, we compared the number of miRNA-binding sites normalized by number of peaks, for each dataset, between the original miRNA sequences and five negative controls (shuffles). The results are shown in Figure 5D and in Supplemental Figures S16D, S17D, S18D, S19D and S20D. Overall, we identified a statistically higher number of miRNA-binding sites per peak in the original datasets compared to each negative control for miRBShunter, TargetScan and miRanda and for both *J*20 threshold and the merge method (*P*< 0.05, Wilcoxon signed-rank test). The same analysis was performed by filtering out the multiple interactions on the promiscuous binding sites and, therefore, considering each promiscuous binding site as one, to avoid the bias toward those miRNAs that share similar sequences and tend to target the same binding sites in the reference datasets, but likely not in the shuffle controls. As shown in the Supplemental Figure S21, we also identified a statistically higher number of miRNA-binding sites per peak in the original datasets in both *J*20 and merge methods for miRBShunter and miRanda. However, TargetScan did not show any difference between reference and shuffle datasets and mainly found one binding site per peak. Thus, we concluded that only miRBShunter and miRanda specifically and systematically found multiple binding sites. However, TargetScan that applies a much more stringent algorithm mainly found one miRNA-binding site per peak. These data indicate that the identification of multiple miRNA-binding sites was specific and not by chance.

Together, these data indicate that on the one hand many Ago2 peaks contain multiple miRNA-binding sites that can potentially interplay to synergistically enhance the miRNA-dependent repression. On the other hand, the complexity of the miRNA-dependent silencing of target mRNA is enhanced by the fact that some binding sites can be potentially targeted by many different miRNAs.

### miRgame: a quantitative model to calculate the degree of occupancy of Ago2 CLIP-seq peaks

To study how the miRNA-binding sites within each Ago2 peak interplay to finely control miRNA-dependent silencing, we derived a model to quantify a miRNA degree of occupancy for each peak. We hypothesize that the degree of occupancy could provide a stratification of Ago2 peaks that would positively correlate to miRNA-dependent mRNA repression.

**Figure 6.**
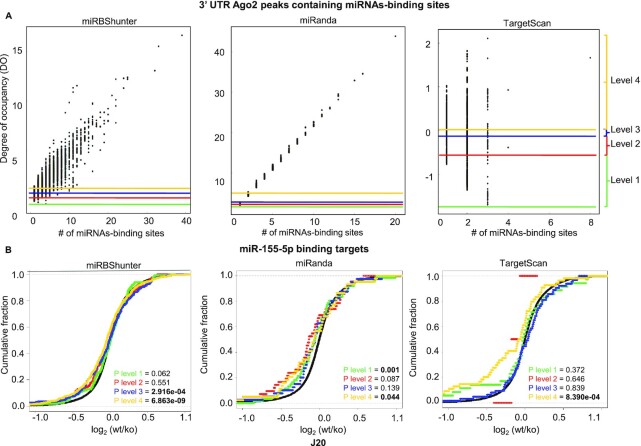
A quantitative model to stratify Ago2 peaks. (**A**) Distributions of the miRNA degree of occupancy by the number of miRNA-binding sites per Ago2 peak in the 3′UTR for miRBShunter, miRanda and TargetScan on the *J*20 threshold peaks. The degree of occupancy calculated on the eleven Ago2 CLIP-seq datasets was divided into four levels depending on quartiles. Levels are highlighted by different colors, as indicated on the right side of the panel. (**B**) Cumulative distributions of miRBShunter, miRanda and TargetScan on the *J*20 threshold peaks showing the target repression upon mmu-miR-155-5p knockout in mouse T-cells for the four levels of degree of occupancy. For each level, we calculated the *P*-value by the Kolmogorov−Smirnov statistical test for target downregulation compared to background (cumulative distribution of all genes).

We calculated the degree of occupancy for all peaks containing miRNA-binding sites identified by optiCLIP framework on the 11 Ago2 CLIP-seq datasets of interest and using two additional prediction programs, namely, miRanda and TargetScan, to compare these data. We found a linear distribution of the degree of occupancy for peaks located only in 3′UTR containing miRNA-binding sites when plotted as a function of the number of miRNA-binding sites within each peak (Figure 6A for *J*20 threshold and Supplemental Figure S22A for the merge method). We also calculated the median of the distance of multiple miRNA-binding sites, but not overlapping, in each peak and found different distances depending on *J*20 threshold or merge methods and the predictor used (Supplemental Figure S23). According to miRgame algorithm, peaks with few miRNA-binding sites or high free energy of the miRNA::RNA binding have lower values of degree of occupancy compared to those with several binding sites that have higher levels. Accordingly, the few multiple binding sites containing peaks predicted by TargetScan showed higher degree of occupancy.

To assess whether the degree of occupancy could be useful to stratify Ago2 CLIP-seq peaks for correlation with target repression, we divided it into four levels based on 3′UTR peak quartiles of the degree of occupancy for each prediction program (Figure 6A). Then, we calculated the significance of the downregulation in each level for the mRNAs containing binding sites for mmu-miR-155-5p, as our best miRNA-mediated repression dataset compared to the other four miRNAs (Figure 4 and Supplemental Figures S14 and S15). The results are presented in Figure 6B for *J*20 threshold and in Supplemental Figure S22B for the merge method. Concerning the *J*20 threshold, we found that levels three and four have the strongest target repression for predictions performed by miRBShunter, while for miRanda and TargetScan level four was overall linked with strongest target repression. Similar results were found for the merge method, except for TargetScan that does not show any target repression. We also calculated the significance of the downregulation in each level for the mRNAs containing binding sites for hsa-miR-16-5p, as our second-best miRNA-mediated repression dataset (Figure 4 and Supplemental Figures S14 and S15). As shown in Supplemental Table S3, for this miRNA useful stratification was observed for miRanda merge. For TargetScan *J*20 significant repression was observed in the level 3. In this latest condition, we further divided the peaks in 2 levels of occupancy degree instead of 4 and found that significant repression was observed in the highest level.

In addition, for mmu-miR-155-5p, we compared the repression calculated by miRgame with that by stratifying the dataset with simple counting of miRNA-binding sites per peak (Supplemental Table S4). This analysis indicates that the simple-count method yields similar results of miRgame for miRBShunter and miRanda. However, it fails to stratify peaks according to the degree of repression for TargetScan. these results suggest that miRgame outperforms when used together with TargetScan *J*20. We then repeated the analyses by separating single binding sites peaks and in multiple binding sites peaks (Supplemental Table S5). Briefly, we found that when considered solely peaks containing one miRNA-binding site, only TargetScan *J*20 associated with miRgame provides a peak stratification that positively correlates with target repression. However, considering solely peaks having multiple binding sites, both miRBShunter and miRanda provide a stratification that positively correlates with target repression, but not TargetScan. Therefore, these analyses indicate that miRgame is providing specific peak stratification when used with TargetScan *J*20, by taking into account binding properties of miRNA::RNA heteroduplexes and the number of miRNA-binding sites within each peak. Whereas, using miRBShunter and miRanda would offer a peak stratification based on multiple binding sites that could synergistically cooperate to repress gene expression.

Altogether, these results overall indicate interdependence between the miRNA-dependent downregulation and high score of the degree of occupancy.

## DISCUSSION

A major goal of systems biology is to develop appropriate computational models that enable the integration of different experimental data to decipher the biological complexity in gene expression regulation. Nowadays, high-throughput sequencing associated to biological experiments is widely used by researchers, thus there is a need for optimized computational pipelines to standardize the data analysis. To gain insights into the global impact of miRNA targeting in post-transcriptional control of gene expression, here we combined a computational method to identify miRNA-binding sequences from Ago2 CLIP-seq data and RNA profiles. We developed a new benchmarked framework, called optiCLIP, to find high-confidence miRNA-binding sites from Ago CLIP-seq data. To the best of our knowledge, this is the first bioinformatics framework developed specifically for Ago CLIP-seq data processing and analysis. OptiCLIP employs benchmarked tools in order to extract reproducible data to gain insights into the miRNA mode of binding. Within optiCLIP, we developed a novel method that addresses the reproducibility of the peaks and integrated a recently developed method to identify miRNA-binding sites that do not exclusively use perfect seed-match. To investigate the validity and efficiency of our new framework, we systematically applied optiCLIP to eleven Ago2 CLIP-seq datasets from mouse and human samples and identified 98,999 miRNA-binding sites.

Briefly, the new method we use in optiCLIP consists in performing the peak calling step in each replicate separately and then quantify the overlap of peaks among multiple replicates through the Jaccard index (J-index) calculation. The consensus peaks are provided by the union of all the peaks that fulfill the reproducibility constrains. We found that 20% of the Jaccard index threshold outperformed other more stringent thresholds, namely, 40% and 80%, in terms of number of identified Ago2 peaks, number of miRNA-binding sites identified in the 3′UTR, and the percentage of peaks containing miRNA-binding sites.

Furthermore, *J*20 threshold outperformed the merge method in terms of peaks and miRNA-binding sites enriched in 3′UTR, however, the merge method was always more sensitive. Together these results would suggest that the *J*20 threshold may be more accurate to predict functional miRNA-binding sites compared to the merge method. These data were validated using different prediction programs, namely, miRBShunter, TargetScan, miRanda, TarPmiR and RNAhybrid. In our analysis, we found that miRBShunter, TargetScan, and miRanda outperformed TarPmiR and RNAhybrid to predict miRNA targets with statistically significant repression. In particular, TargetScan associated with the *J*20 threshold predicted miRNA targets that showed the best repression, however limited to the perfect seed-match search to the 3′UTR. miRanda, which uses similar features that TargetScan, is less stringent and thus would allow a more complete identification of miRNA-binding sites beyond the 3′UTR. However, it would include more false positives than TargetScan. Finally, miRBShunter predicts both canonical and non-canonical miRNA-binding sites on all transcriptomic regions, by identifying all potential miRNA::RNA heteroduplexes for both seed and non-seed mode of binding based on the enriched motif sequences on Ago peaks. In addition, because less stringent than TargetScan, miRanda and miRBShunter predict the presence of multiple binding sites that could synergistically cooperate by providing, therefore, a more complete miRNA-dependent gene expression program. Thus, depending on their own findings, results, and rationale, scientists can choose their own favorite program(s) that would better fit to their research.

We characterized the binding preferences of the most expressed miRNAs, revealing binding patterns that involve the seed and outside the seed bindings. These results are due to the intrinsic assumption of the miRBShunter that looks for enriched motifs in the target sequences with no restrictions in terms of mode of binding. The diversity of the binding preference among the miRNAs can be explained by the fact that each miRNA has its own specific sequence, which can influence the efficiency of the pairing composition. This conclusion is supported by the fact that miRNAs belonging to the same family show similar binding preferences (38).

As previously described (4,6,9,36), we also found that miRNA-binding sites are located in different parts of the transcriptome, especially in the 3′UTR and the CDS. Moreover, we found that 3′UTR miRNA-binding sites identified by optiCLIP can actually trigger significant target downregulation in a miRNA-specific fashion. In particular, for targets identified by optiCLIP, significant repression over the background was shown for mmu-miR-155-5p, mmu-let-7a-5p and hsa-miR-124-3p. Overall, we also noticed that for *J*20, even if a smaller number of targets was identified for all prediction programs compared to the merge method, they were significantly more repressed of those identified by the merge method. These data indicate that *J*20 threshold once again outperformed the merge method by filtering out false positive peaks.

Finally, we set up a quantitative model employing a game theory approach to calculate by a newly developed algorithm the degree of occupancy of miRNA binding for each Ago2 peak. The degree of occupancy allows the stratification of Ago2 peaks that correlates with the target repression. We called this novel method the miRgame. Importantly, the degree of occupancy positively correlates with the number of binding sites within the peak and a stronger target repression. These results are in line with recent experimental and computational evidence (30,34,39,40) and indicate that miRNA cooperation within the Ago2 peaks is an important feature by which specificity is gained in the silencing of the targets. Although longer distances were not tested, synergistic cooperation between two miRNA-binding sites is effective within a distance of 60 nt (41). Different factors that might explain the different distance of cooperative miRNA-binding sites found in several publications (30,34,39,40) would include different linker and flanking sequences, as well as the milieu of RNA-binding proteins. On the other hand, the saturation of binding sites targeted by multiple miRNAs (promiscuous miRNA-binding sites) can explain the relatively poor ability that changes of single miRNA expression may modulate target repression, as it was previously suggested (34). In conclusion, the synergistic cooperation of miRNA-binding sites and the presence of promiscuous binding sites within each Ago2 peak would define miRNA-binding units to sophistically regulate target repression.

Compared to other models of miRNA target regulation that consider the mechanism of one or few miRNAs (34,42–45), miRgame is based on the comprehensive identification of the whole set of miRNA targetome by CLIP-seq analysis. By taking into account each miRNA-binding unit, miRgame determines the synergy of multiple binding sites, multiple miRNA targeting to the same binding site, and the energy of binding within each Ago2 peak. Compared to models not based on CLIP-seq data (45), our method is not biased toward the search of seed-match sequence to 3′UTR and target abundance. Our comparative analyses indicate that miRgame stratifies according to the miRNA-binding properties when used with TargetScan *J*20. However, when miRBShunter or miRanda are used, miRgame is providing peak stratification based on multiple binding sites identification that could synergistically cooperate for a stronger repression. Thus, depending on their own findings, results, and rationale, researchers can choose their own favorite program(s) that would better fit to their research.

In summary, here we proposed an optimized bioinformatic framework, called optiCLIP, to analyze Ago2 CLIP-seq datasets, and showed by comparative studies its reliability and robustness in comprehensively identifying miRNA-binding sites. We used a systematic approach based on an adaptation of the game theory, to propose a quantitative model for stratification of Ago2 CLIP-seq peaks, which revealed that the synergy among miRNA-binding sites in each Ago2 peak finely controls target repression. This model can be also used for *in silico* stratification of predicted miRNA-binding sites. In conclusion, with increasing number of high-throughput data for miRNA targeting, our method is providing a powerful tool to interpret the dynamics and the complexity of miRNA-dependent post-transcriptional gene expression. In the future, it should be possible to extend our model to integrate post-transcriptional regulation by other regulators, including RNA-binding proteins that regulate miRNA targeting, such as HuR (46), Dnd1 (47) and Sfpq (36), RNA folding, and mRNA expression levels. Our method will help researchers to better understand the miRNA mode of action involved in complex biological processes.

## DATA AVAILABILITY

Raw data for Ago2 CLIP-seq datasets and microarray were downloaded from GEO database or specific websites (Tables 1 and 2).

## Supplementary Material

gkab198_Supplemental_Files

## Notes

Present address: Silvia Bottini, Université Côte d’Azur, Center of Modeling, Simulation and Interactions, Nice, France.
