# Supplementary material for: Systemic CLIP-seq analysis and game theory approach to model microRNA mode of binding
Source: Nucleic Acids Res. 2021 Apr 6;49(11):e66. doi: 10.1093/nar/gkab198 (PMC8216473; doi:10.1093/nar/gkab198)
Supplement: gkab198_Supplemental_Files [file gkab198_supplemental_files.zip › new Supplemental figures legend.docx]

**Supplemental figures legend**

**Supplemental Figure 1.** optiCLIP is a computational framework to analyze Ago2 CLIP-seq experiments from raw data to the identification of miRNA-binding sites.

**Supplemental Figure 2.** Barplots reporting the transcriptomic location of the miRNA-binding sites identified by (A) miRanda, (B) RNAhybrid and (C) TarPmiR for the indicated methods to identify peaks. Percentage of peaks containing miRNA-binding sites identified by miRBShunter (D) and miRanda (E) on peaks identified by different J-index thresholds and the merge method for the indicated datasets.

**Supplemental Figure 3.** (A) Heatmap showing the miRNA mode of binding in the human datasets. miRNAs are grouped by hierarchical clustering using “hclust” by R software. The color intensity (scale range from 0 to 1) represents the normalized frequency for each miRNA nucleotide position. (B) K-means dendrogram with group colors showing the clusterization of human miRNAs. The clustering analyses were performed on miRNA-binding sites identified by miRBShunter on Ago2 peaks found using the merge method.

**Supplemental Figure 4.** (A) Heatmap showing the miRNA mode of binding in the human datasets. miRNAs are grouped by hierarchical clustering using “hclust” by R software. The color intensity (scale range from 0 to 1) represents the normalized frequency for each miRNA nucleotide position. (B) K-means dendrogram with group colors showing the clusterization of human miRNAs. The clustering analyses were performed on miRNA-binding sites identified by TargetScan on Ago2 peaks found using the J20 threshold.

**Supplemental Figure 5.** (A) Heatmap showing the miRNA mode of binding in the human datasets. miRNAs are grouped by hierarchical clustering using “hclust” by R software. The color intensity (scale range from 0 to 1) represents the normalized frequency for each miRNA nucleotide position. (B) K-means dendrogram with group colors showing the clusterization of human miRNAs. The clustering analyses were performed on miRNA-binding sites identified by TargetScan on Ago2 peaks found using the merge method.

**Supplemental Figure 6.** (A) Heatmap showing the miRNA mode of binding in the human datasets. miRNAs are grouped by hierarchical clustering using “hclust” by R software. The color intensity (scale range from 0 to 1) represents the normalized frequency for each miRNA nucleotide position. (B) K-means dendrogram with group colors showing the clusterization of human miRNAs. The clustering analyses were performed on miRNA-binding sites identified by miRanda on Ago2 peaks found using the J20 threshold.

**Supplemental Figure 7.** (A) Heatmap showing the miRNA mode of binding in the human datasets. miRNAs are grouped by hierarchical clustering using “hclust” by R software. The color intensity (scale range from 0 to 1) represents the normalized frequency for each miRNA nucleotide position. (B) K-means dendrogram with group colors showing the clusterization of human miRNAs. The clustering analyses were performed on miRNA-binding sites identified by miRanda on Ago2 peaks found using the merge method.

**Supplemental Figure 8.** (A) Heatmap showing the miRNA mode of binding in the mouse datasets. miRNAs are grouped by hierarchical clustering using “hclust” by R software. The color intensity (scale range from 0 to 1) represents the normalized frequency for each miRNA nucleotide position. (B) K-means dendrogram with group colors showing the clusterization of human miRNAs. The clustering analyses were performed on miRNA-binding sites identified by miRBShunter on Ago2 peaks found using the J20 threshold.

**Supplemental Figure 9.** (A) Heatmap showing the miRNA mode of binding in the mouse datasets. miRNAs are grouped by hierarchical clustering using “hclust” by R software. The color intensity (scale range from 0 to 1) represents the normalized frequency for each miRNA nucleotide position. (B) K-means dendrogram with group colors showing the clusterization of human miRNAs. The clustering analyses were performed on miRNA-binding sites identified by miRBShunter on Ago2 peaks found using the merge method.

**Supplemental Figure 10.** (A) Heatmap showing the miRNA mode of binding in the mouse datasets. miRNAs are grouped by hierarchical clustering using “hclust” by R software. The color intensity (scale range from 0 to 1) represents the normalized frequency for each miRNA nucleotide position. (B) K-means dendrogram with group colors showing the clusterization of human miRNAs. The clustering analyses were performed on miRNA-binding sites identified by TargetScan on Ago2 peaks found using the J20 threshold.

**Supplemental Figure 11.** (A) Heatmap showing the miRNA mode of binding in the mouse datasets. miRNAs are grouped by hierarchical clustering using “hclust” by R software. The color intensity (scale range from 0 to 1) represents the normalized frequency for each miRNA nucleotide position. (B) K-means dendrogram with group colors showing the clusterization of human miRNAs. The clustering analyses were performed on miRNA-binding sites identified by TargetScan on Ago2 peaks found using the merge method.

**Supplemental Figure 12.** (A) Heatmap showing the miRNA mode of binding in the mouse datasets. miRNAs are grouped by hierarchical clustering using “hclust” by R software. The color intensity (scale range from 0 to 1) represents the normalized frequency for each miRNA nucleotide position. (B) K-means dendrogram with group colors showing the clusterization of human miRNAs. The clustering analyses were performed on miRNA-binding sites identified by miRanda on Ago2 peaks found using the J20 threshold.

**Supplemental Figure 13.** (A) Heatmap showing the miRNA mode of binding in the mouse datasets. miRNAs are grouped by hierarchical clustering using “hclust” by R software. The color intensity (scale range from 0 to 1) represents the normalized frequency for each miRNA nucleotide position. (B) K-means dendrogram with group colors showing the clusterization of human miRNAs. The clustering analyses were performed on miRNA-binding sites identified by miRanda on Ago2 peaks found using the merge method.

**Supplemental Figure 14.** Cumulative distributions showing target repression upon miRNA overexpression of (A) mmu-let-7a-5p, (B) hsa-miR-1-3p, (C) hsa-miR-16-5p for the target RNAs identified with the indicated prediction programs. P-value of target repression was calculated using Kolmogorov-Smirnov statistical test.

**Supplemental Figure 15.** Cumulative distributions showing target repression upon miRNA overexpression of hsa-miR-124-3p for the target RNAs identified with the indicated prediction programs. P-value of target repression was calculated using Kolmogorov-Smirnov statistical test.

**Supplemental Figure 16.** Heatmaps showing (A) the percentage of peaks containing single and multiple miRNA-binding sites and (B) percentage of peaks containing promiscuous miRNA-binding sites identified by miRBShunter using the merge method for the indicated Ago2 CLIP-seq datasets. (C) Plot showing the significant correlation (Spearman rho: 0.899) between the single/multiple and the promiscuous miRNA-binding sites identified by miRBShunter using the merge method (D) Boxplot showing the number of miRNA-binding sites identified by miRBShunter on peaks identified by the merge method, normalized for the number of peaks, on the indicated datasets. Wilcoxon signed-rank test was applied to compare reference with each shuffle. P < 0.05 (*), P < 0.01 (**) and P < 0.001 (***); not significant (ns).

**Supplemental Figure 17.** Heatmaps showing (A) the percentage of peaks containing single and multiple miRNA-binding sites and (B) percentage of peaks containing promiscuous miRNA-binding sites identified by miRanda using the J20 threshold method for the indicated Ago2 CLIP-seq datasets. (C) Plot showing the significant correlation (Spearman rho: 0.640) between the single/multiple and the promiscuous miRNA-binding sites identified by miRanda using the J20 threshold method (D) Boxplot showing the number of miRNA-binding sites identified by miRanda on peaks identified by the J20 threshold method, normalized for the number of peaks, on the indicated datasets. Wilcoxon signed-rank test was applied to compare reference with each shuffle. P < 0.05 (*), P < 0.01 (**) and P < 0.001 (***); not significant (ns).

**Supplemental Figure 18.** Heatmaps showing (A) the percentage of peaks containing single and multiple miRNA-binding sites and (B) percentage of peaks containing promiscuous miRNA-binding sites identified by miRanda using the merge method for the indicated Ago2 CLIP-seq datasets. (C) Plot showing the significant correlation (Spearman rho: 0.637) between the single/multiple and the promiscuous miRNA-binding sites identified by miRanda using the merge method (D) Boxplot showing the number of miRNA-binding sites identified by miRanda on peaks identified by the merge method, normalized for the number of peaks, on the indicated datasets. Wilcoxon signed-rank test was applied to compare reference with each shuffle. P < 0.05 (*), P < 0.01 (**) and P < 0.001 (***); not significant (ns).

**Supplemental Figure 19.** Heatmaps showing (A) the percentage of peaks containing single and multiple miRNA-binding sites and (B) percentage of peaks containing promiscuous miRNA-binding sites identified by TargetScan using the J20 threshold method for the indicated Ago2 CLIP-seq datasets. (C) Plot showing the significant correlation (Spearman rho: 0.999) between the single/multiple and the promiscuous miRNA-binding sites identified by TargetScan using the J20 threshold method (D) Boxplot showing the number of miRNA-binding sites identified by TargetScan on peaks identified by the J20 threshold method, normalized for the number of peaks, on the indicated datasets. Wilcoxon signed-rank test was applied to compare reference with each shuffle. P < 0.05 (*), P < 0.01 (**) and P < 0.001 (***); not significant (ns).

**Supplemental Figure 20.** (Heatmaps showing (A) the percentage of peaks containing single and multiple miRNA-binding sites and (B) percentage of peaks containing promiscuous miRNA-binding sites identified by TargetScan using the merge method for the indicated Ago2 CLIP-seq datasets. (C) Plot showing the significant correlation (Spearman rho: 0.998) between the single/multiple and the promiscuous miRNA-binding sites identified by TargetScan using the merge method (D) Boxplot showing the number of miRNA-binding sites identified by TargetScan on peaks identified by the merge method, normalized for the number of peaks, on the indicated datasets. Wilcoxon signed-rank test was applied to compare reference with each shuffle. P < 0.05 (*), P < 0.01 (**) and P < 0.001 (***); not significant (ns).

**Supplemental Figure 21.** Boxplots showing the number of miRNA-binding sites normalized for the number of peaks, on the indicated datasets, when filtering out the multiple interactions of miRNAs on the same binding site (promiscuous binding sites). The presented results correspond to (A), (C), (E) miRBShunter, miRanda and TargetScan on peaks identified by the J20 threshold method, respectively, and (B), (D), (F) miRBShunter, miRanda and TargetScan on peaks identified by the merge method, respectively. Wilcoxon signed-rank test was applied to compare reference with each shuffle. P < 0.05 (*), P < 0.01 (**) and P < 0.001 (***); not significant (ns).

**Supplemental Figure 22.** (A) Distributions of the miRNA degree of occupancy by the number of miRNA-binding sites per Ago2 peaks in 3’UTR for miRBShunter, miRanda and TargetScan on the peaks identified by the merge method. The degree of occupancy calculated on the eleven Ago2 CLIP-seq datasets was divided into four levels depending on quartiles. Levels are highlighted by different colours, as indicated on the right side of the panel. (B) Cumulative distributions of miRBShunter, miRanda and TargetScan on the peaks identified by the merge method showing the target repression upon mmu-miR-155-5p knockout for the four levels of degree of occupancy. For each level, we calculated the p-value by the Kolmogorov-Smirnov statistical test for target downregulation compared to background (cumulative distribution of all genes).

**Supplemental Figure 23.** Histograms presenting the distributions and the median values of the distances between non-overlapping binding sites (<7 nt) in each peak for (A), (C), (E) miRBShunter, miRanda and TargetScan on peaks identified by the J20 threshold method, respectively, and (B), (D), (F) miRBShunter, miRanda and TargetScan on peaks identified by the merge method, respectively.
