# Supplementary material for: Systemic CLIP-seq analysis and game theory approach to model microRNA mode of binding
Source: Nucleic Acids Res. 2021 Apr 6;49(11):e66. doi: 10.1093/nar/gkab198 (PMC8216473; doi:10.1093/nar/gkab198)
Supplement: gkab198_Supplemental_Files [file gkab198_supplemental_files.zip › Suppl Figures.pdf]

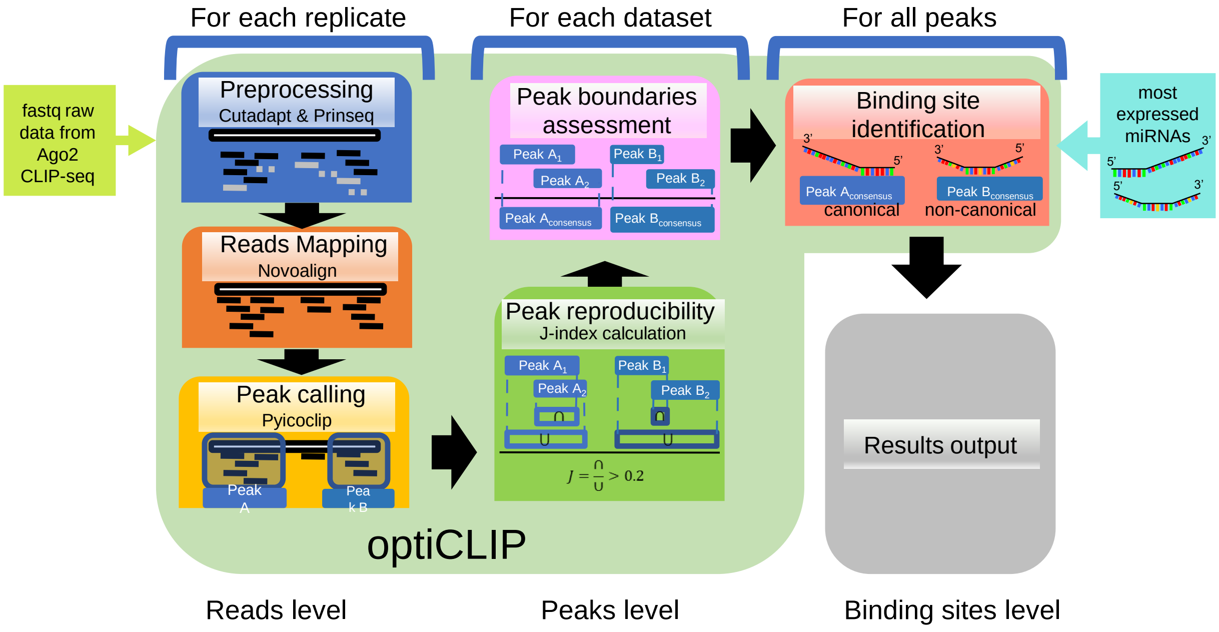

Supplemental Figure 1

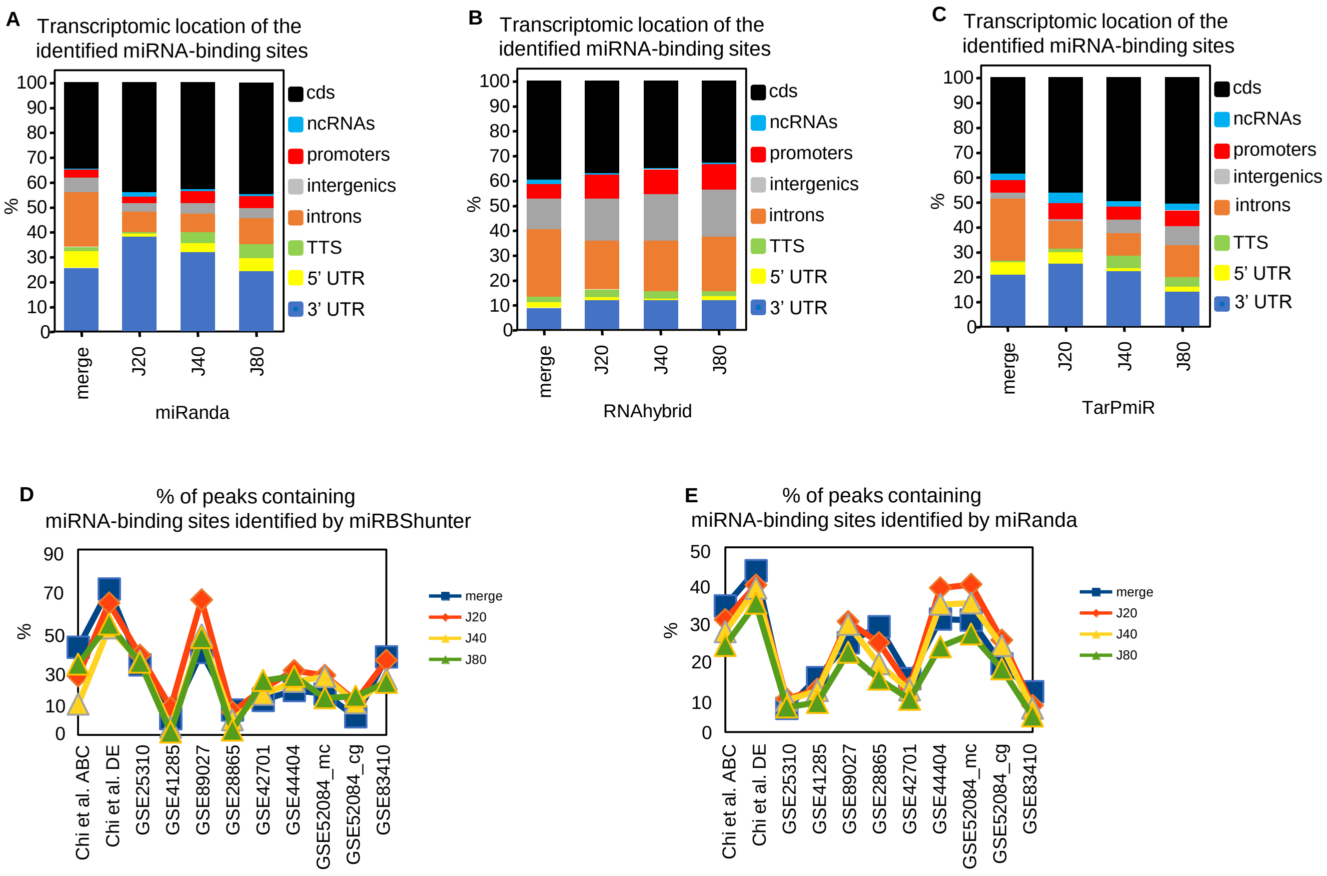

Supplemental Figure 2

Clustering of miRNA-binding sites in human datasets

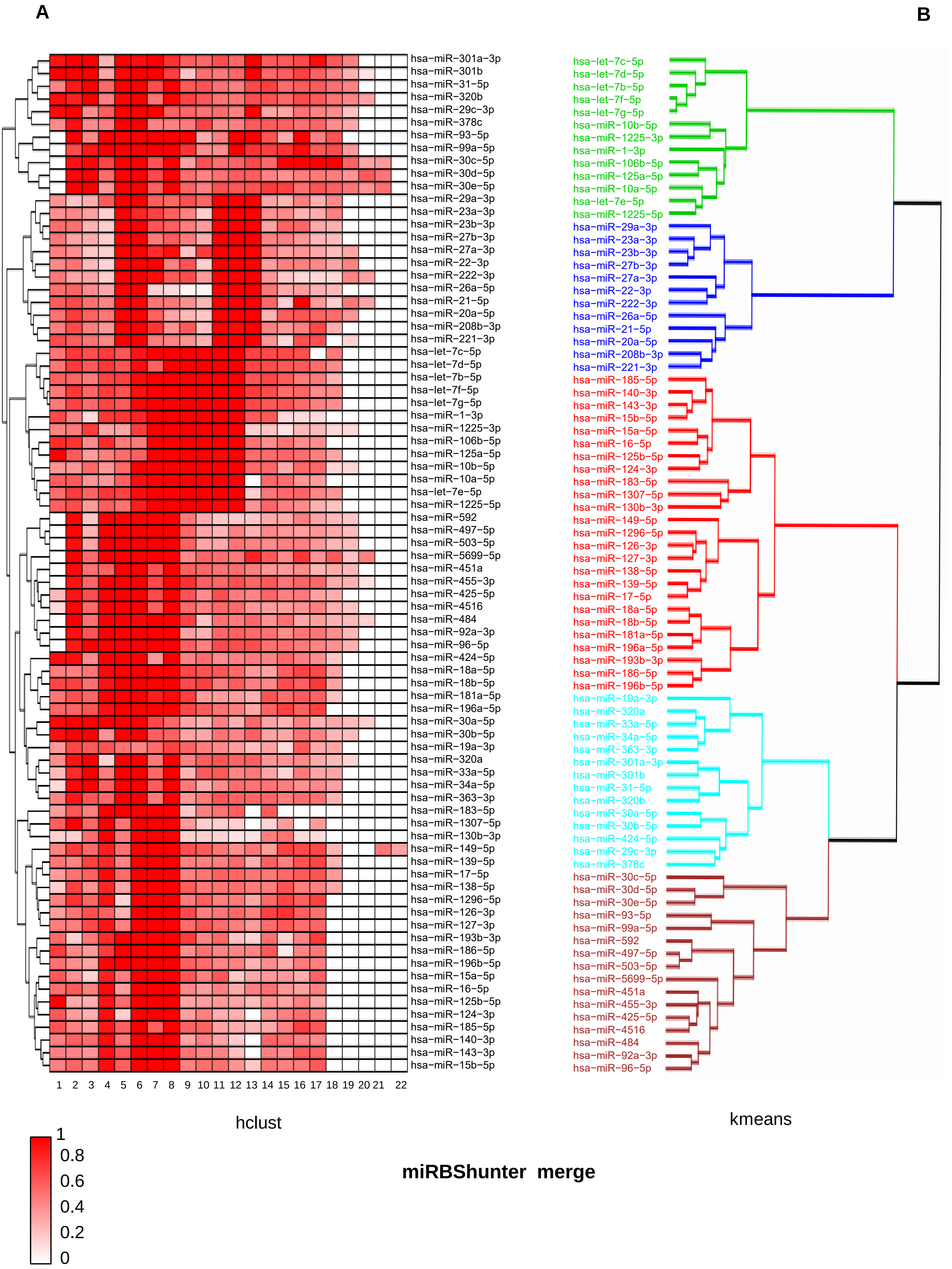

miRBShunter merge

Supplemental Figure 3

Clustering of miRNA-binding sites in human datasets

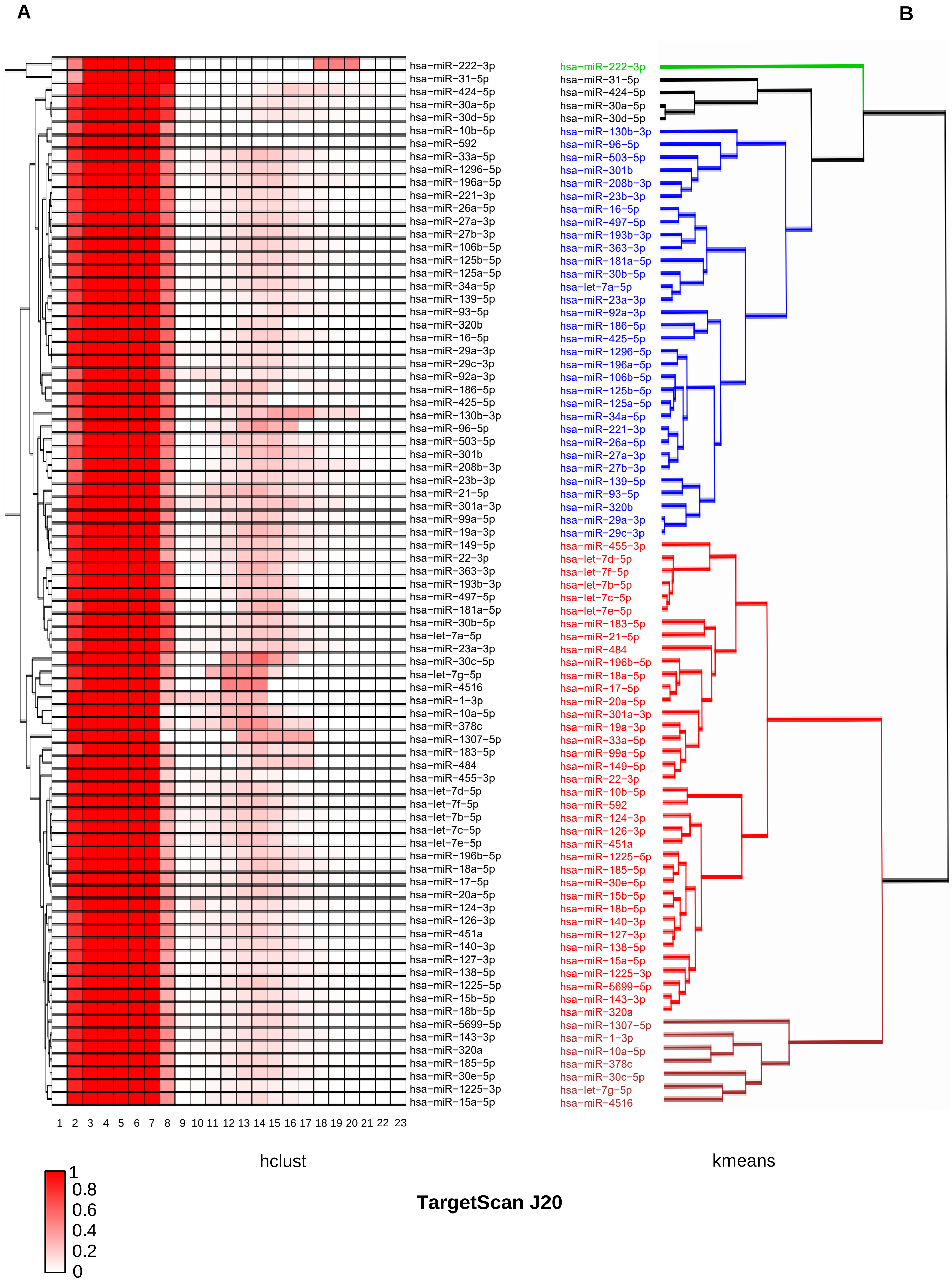

Supplemental Figure 4

Clustering of miRNA-binding sites in human datasets

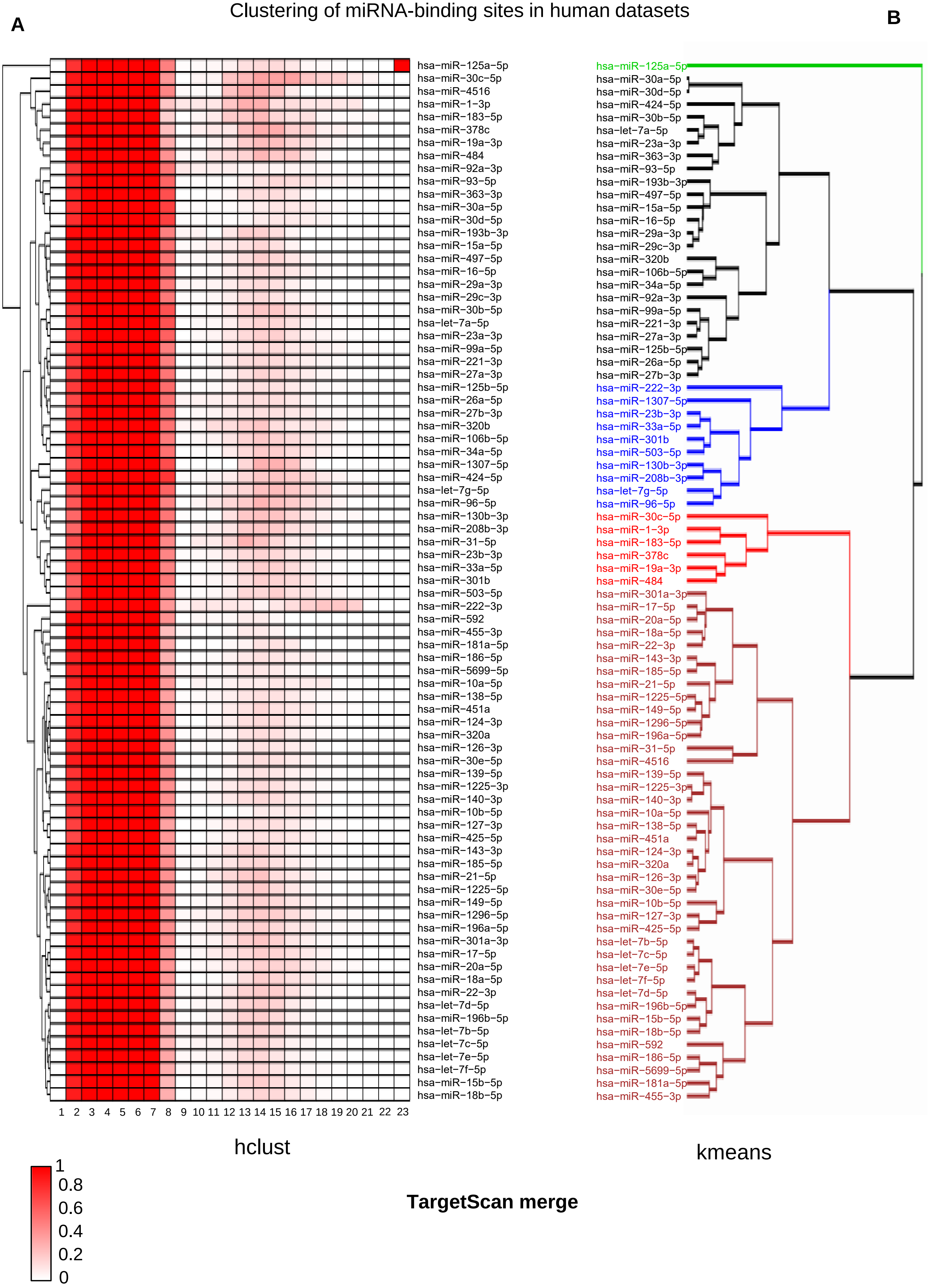

Supplemental Figure 5

Clustering of miRNA-binding sites in human datasets

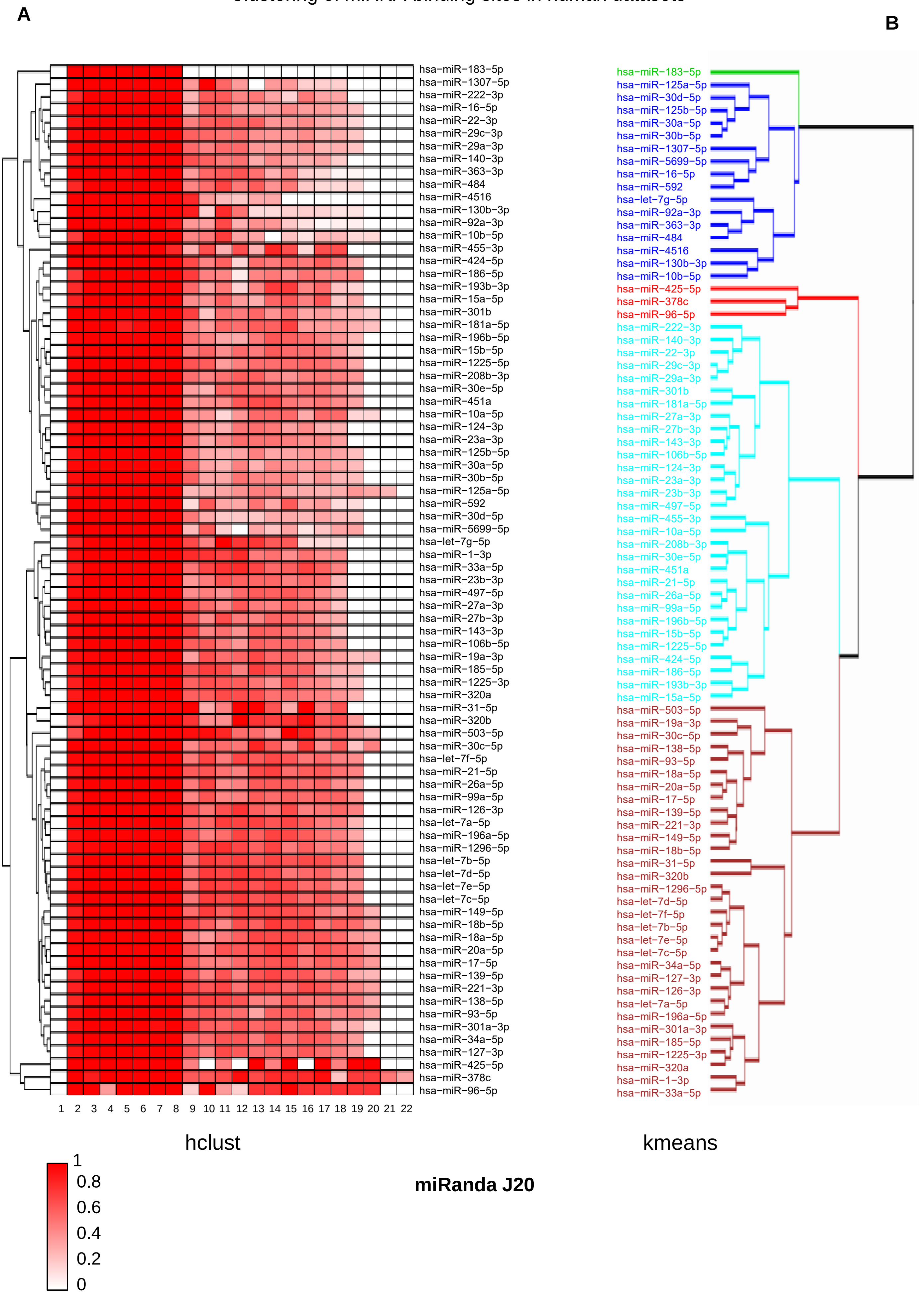

Supplemental Figure 6

Clustering of miRNA-binding sites in human datasets

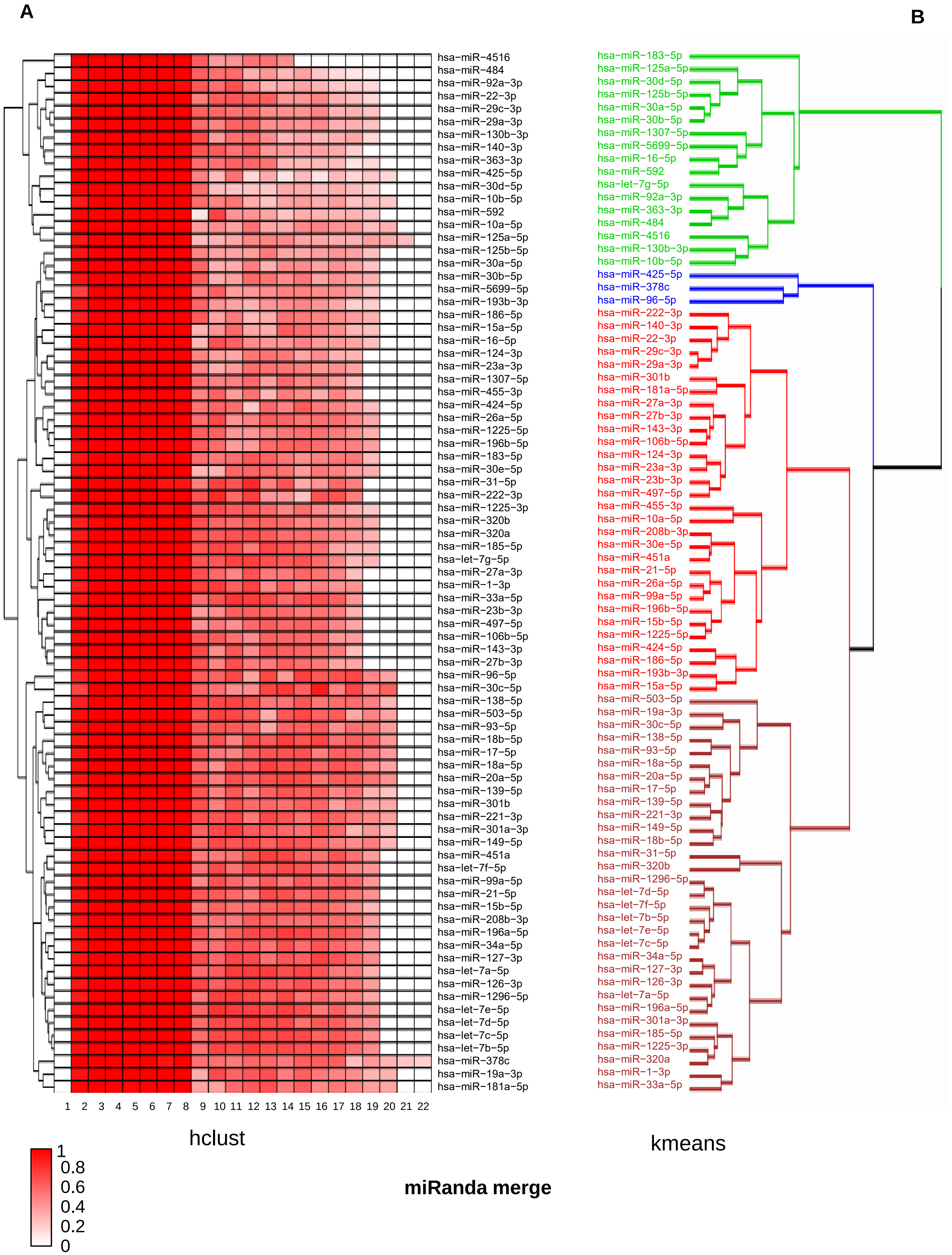

Supplemental Figure 7

Clustering of miRNA-binding sites in mouse datasets

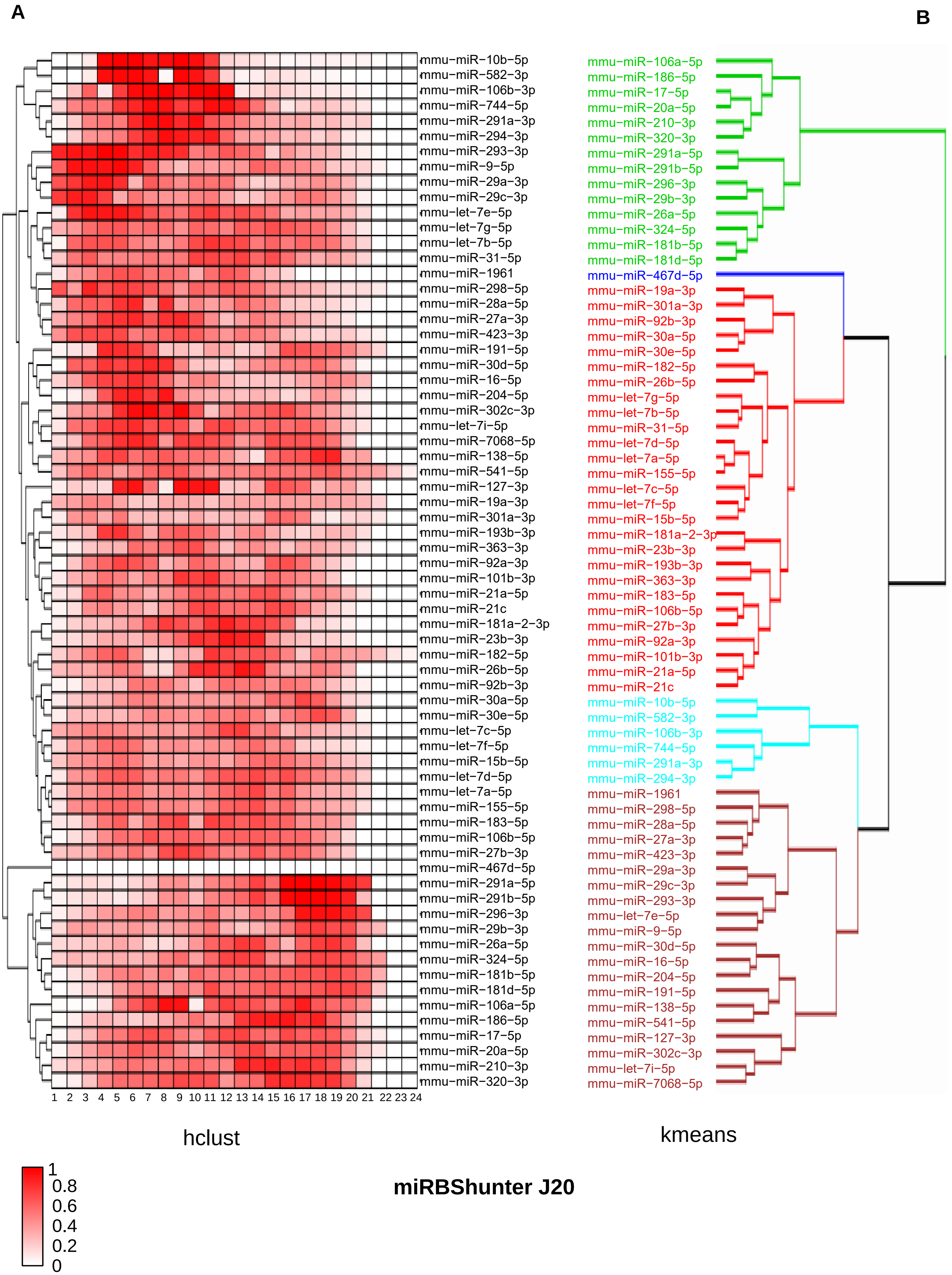

miRBShunter J20

Supplemental Figure 8

Clustering of miRNA-binding sites in mouse datasets

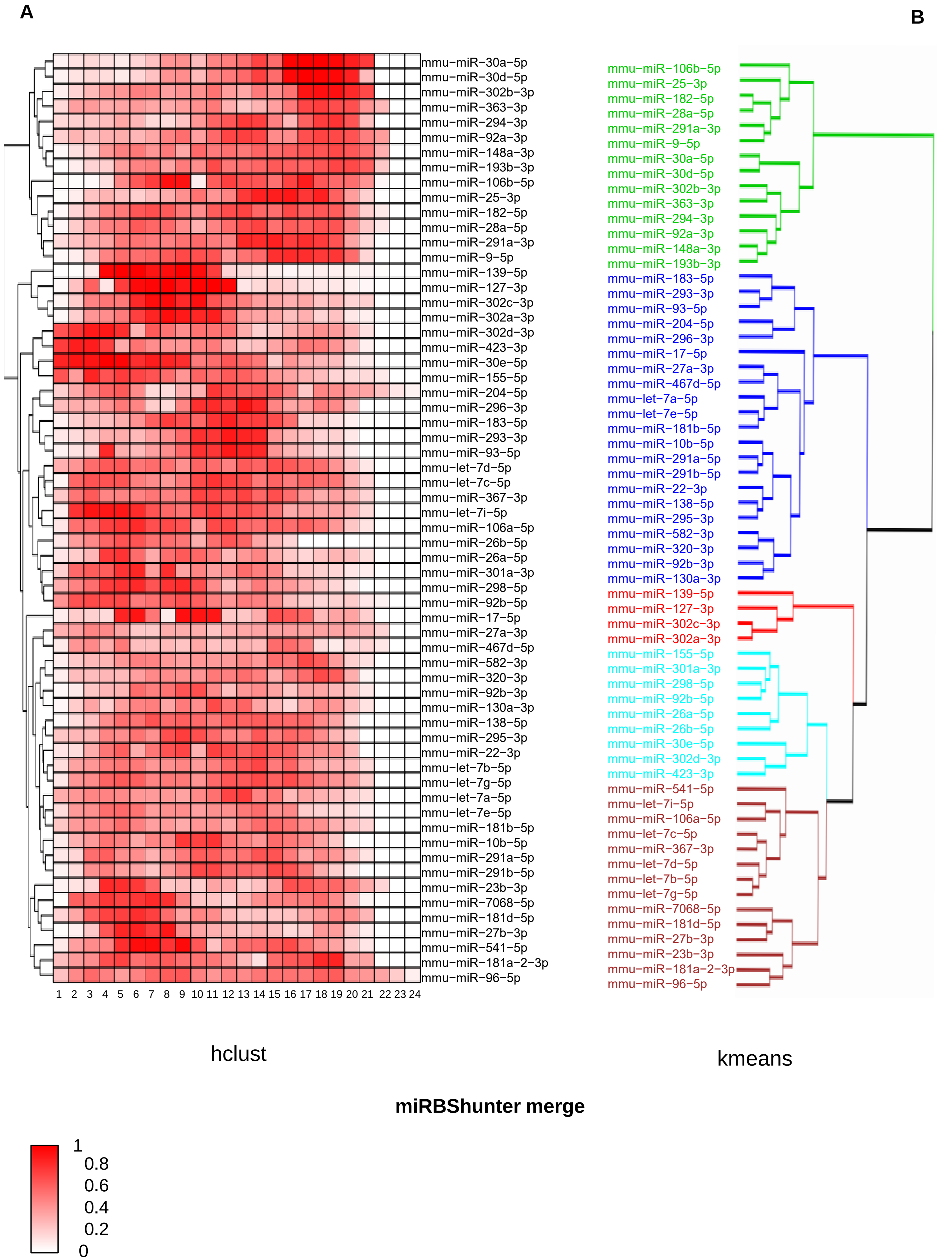

Supplemental Figure 9

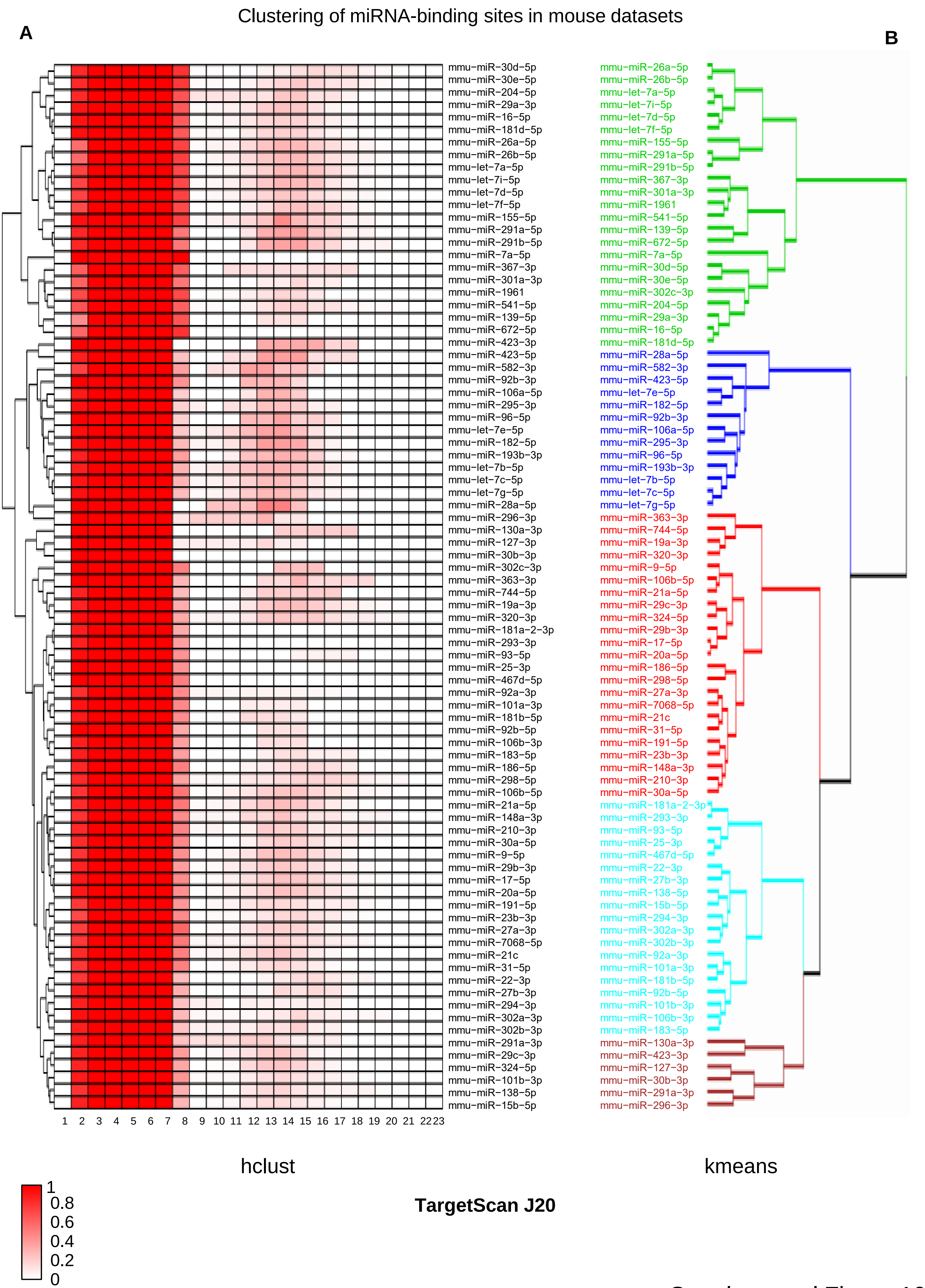

Supplemental Figure 10

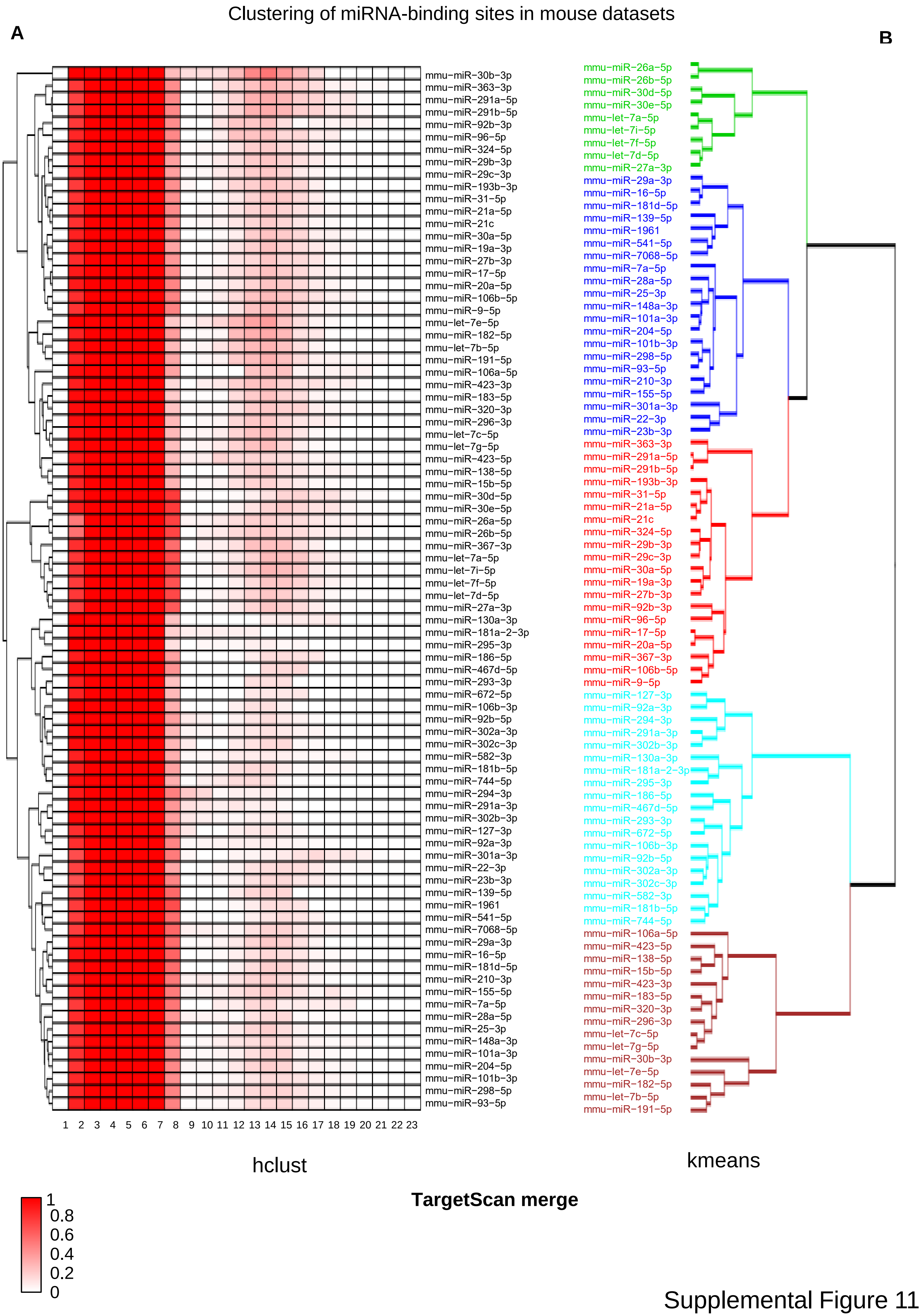

Supplemental Figure 11

Clustering of miRNA-binding sites in mouse datasets

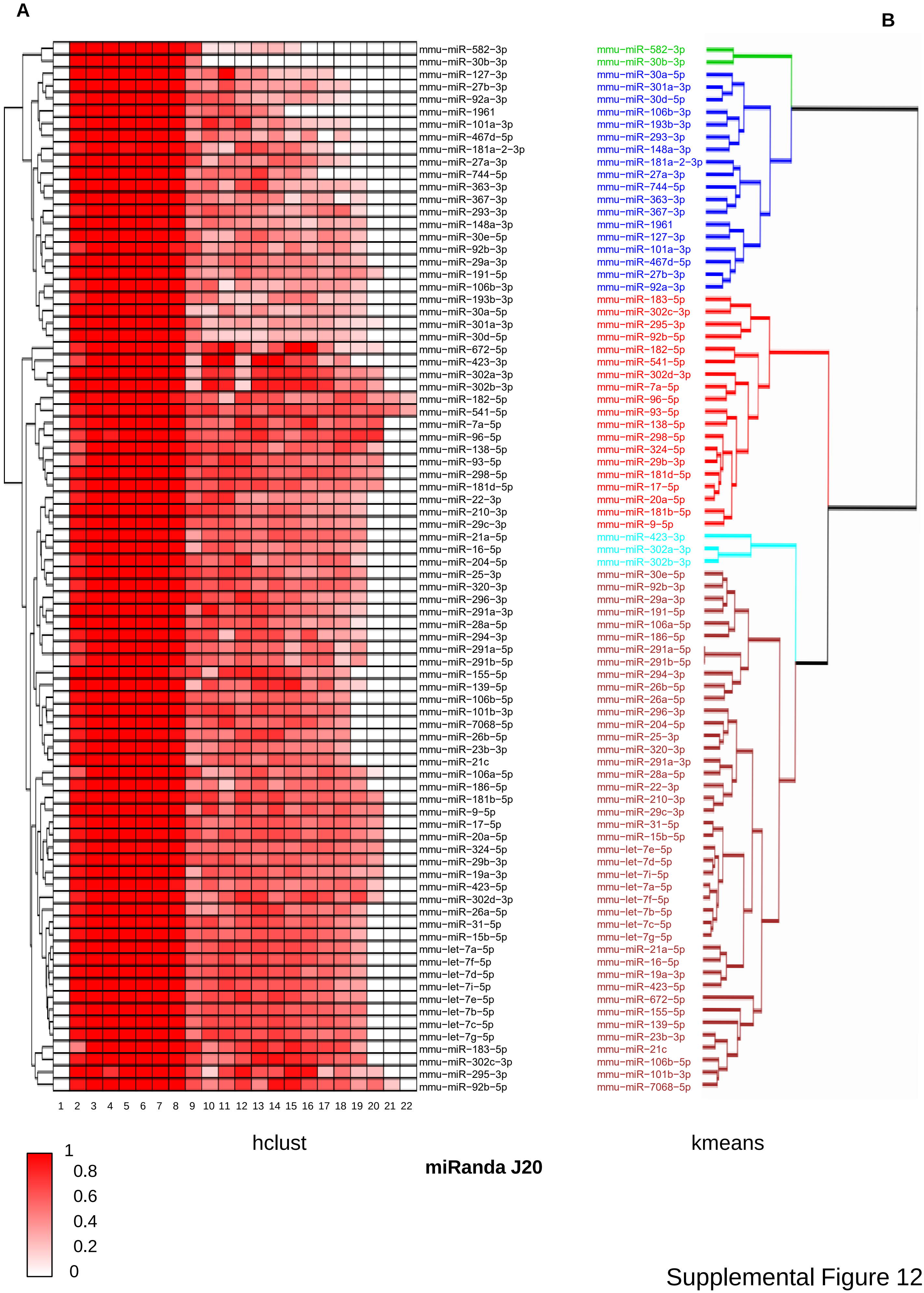

Supplemental Figure 12

Clustering of miRNA-binding sites in mouse datasets

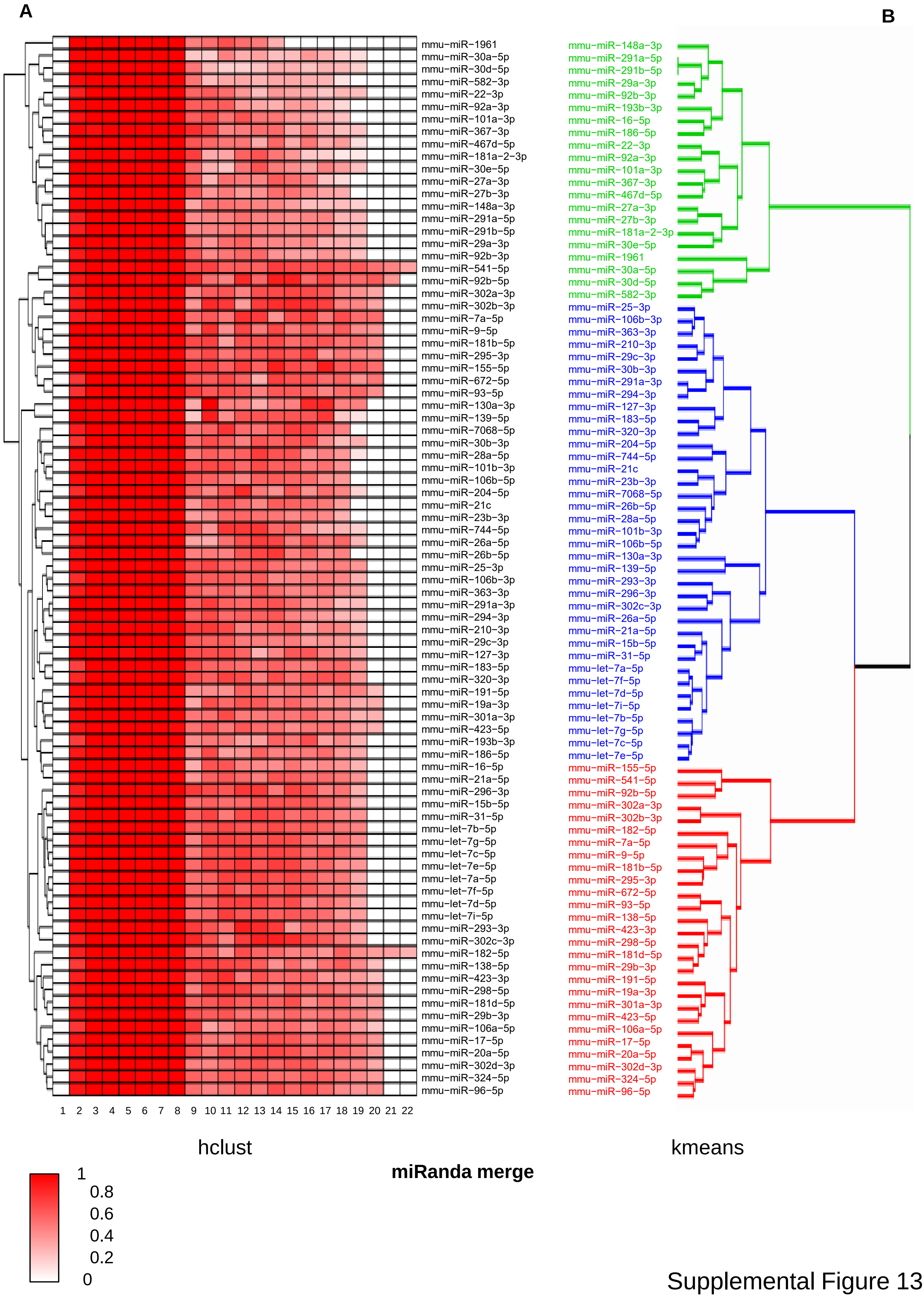

miRanda merge

Supplemental Figure 13

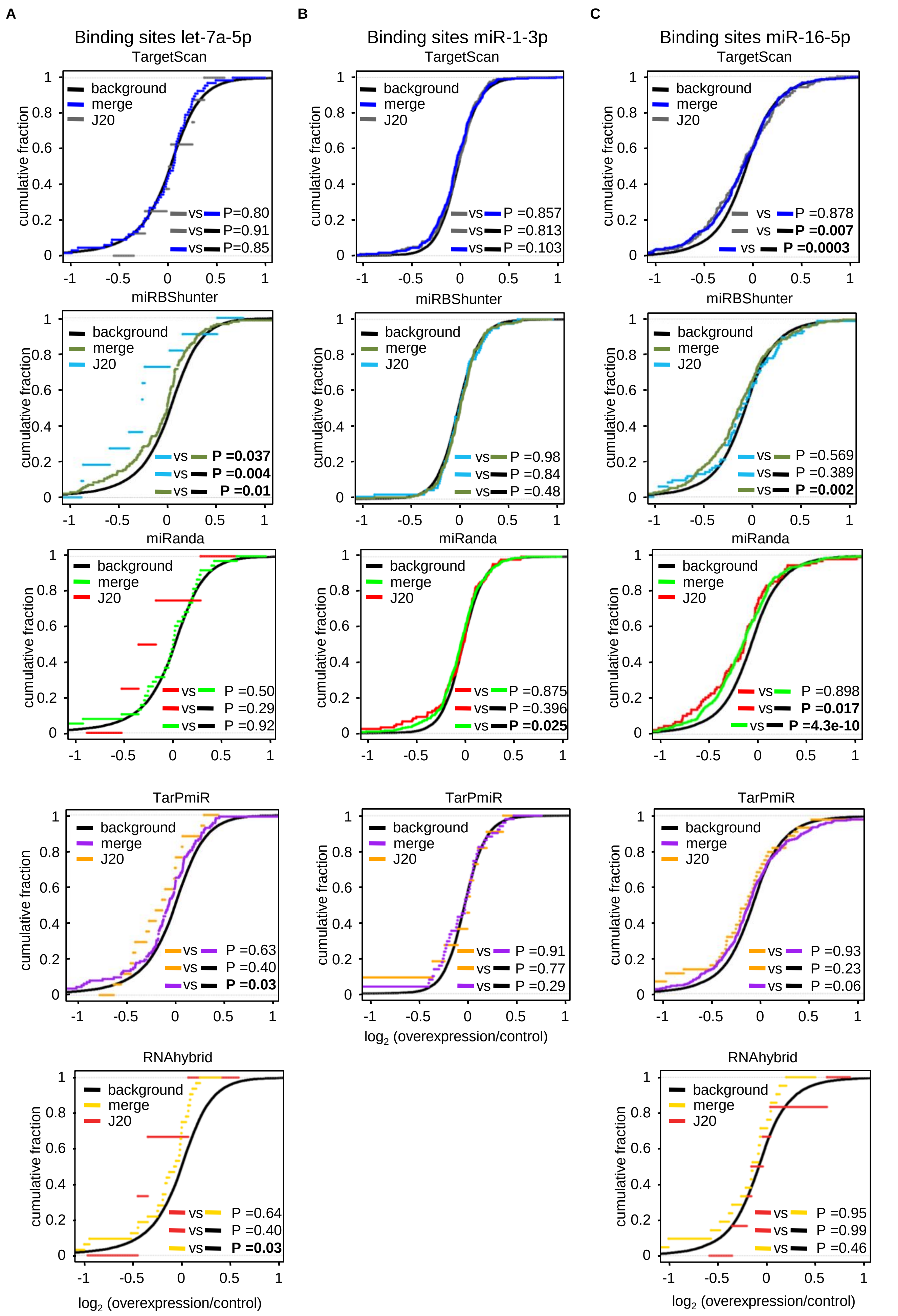

Supplemental Figure 14

Binding sites miR-124-3p

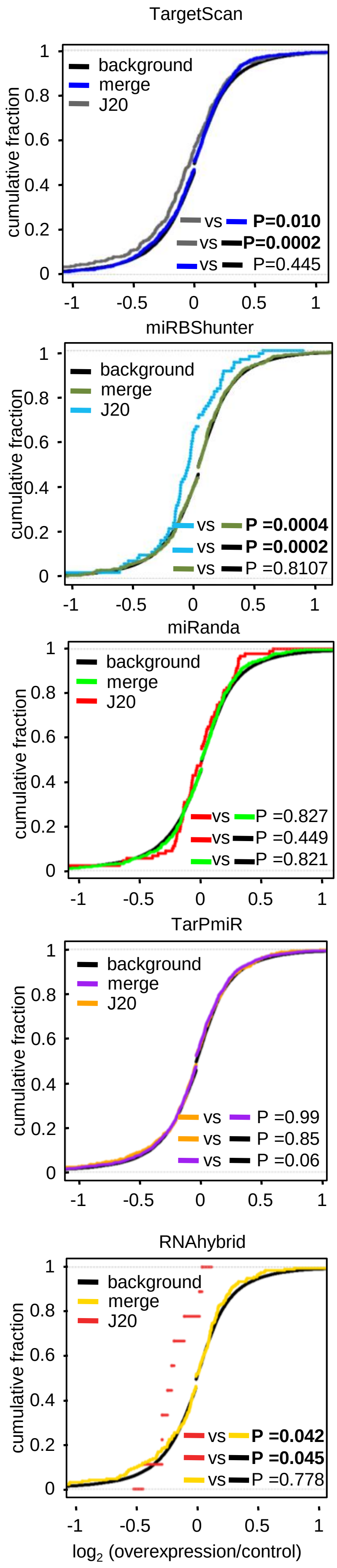

Supplemental Figure 15

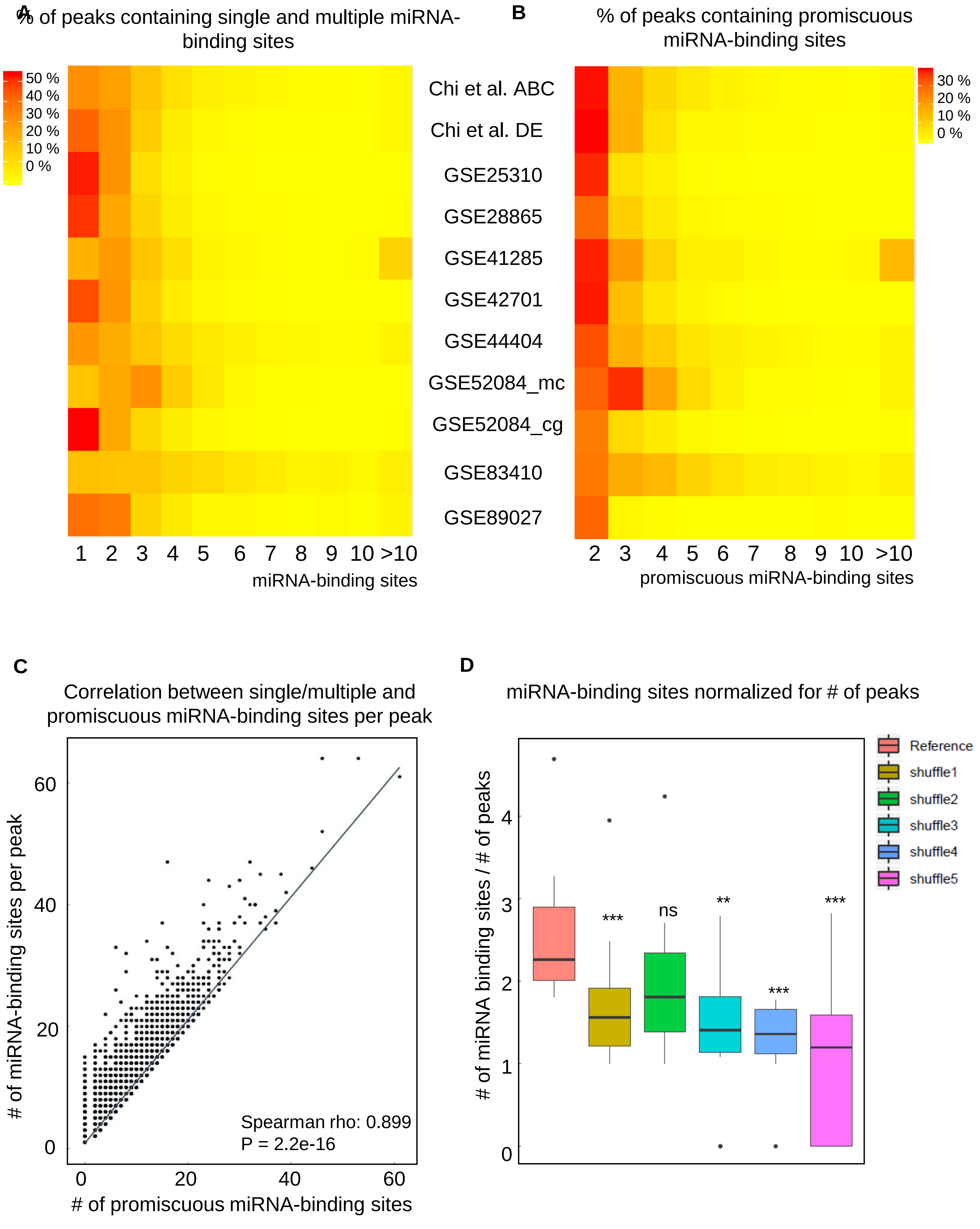

miRBShunter merge

Supplemental Figure 16

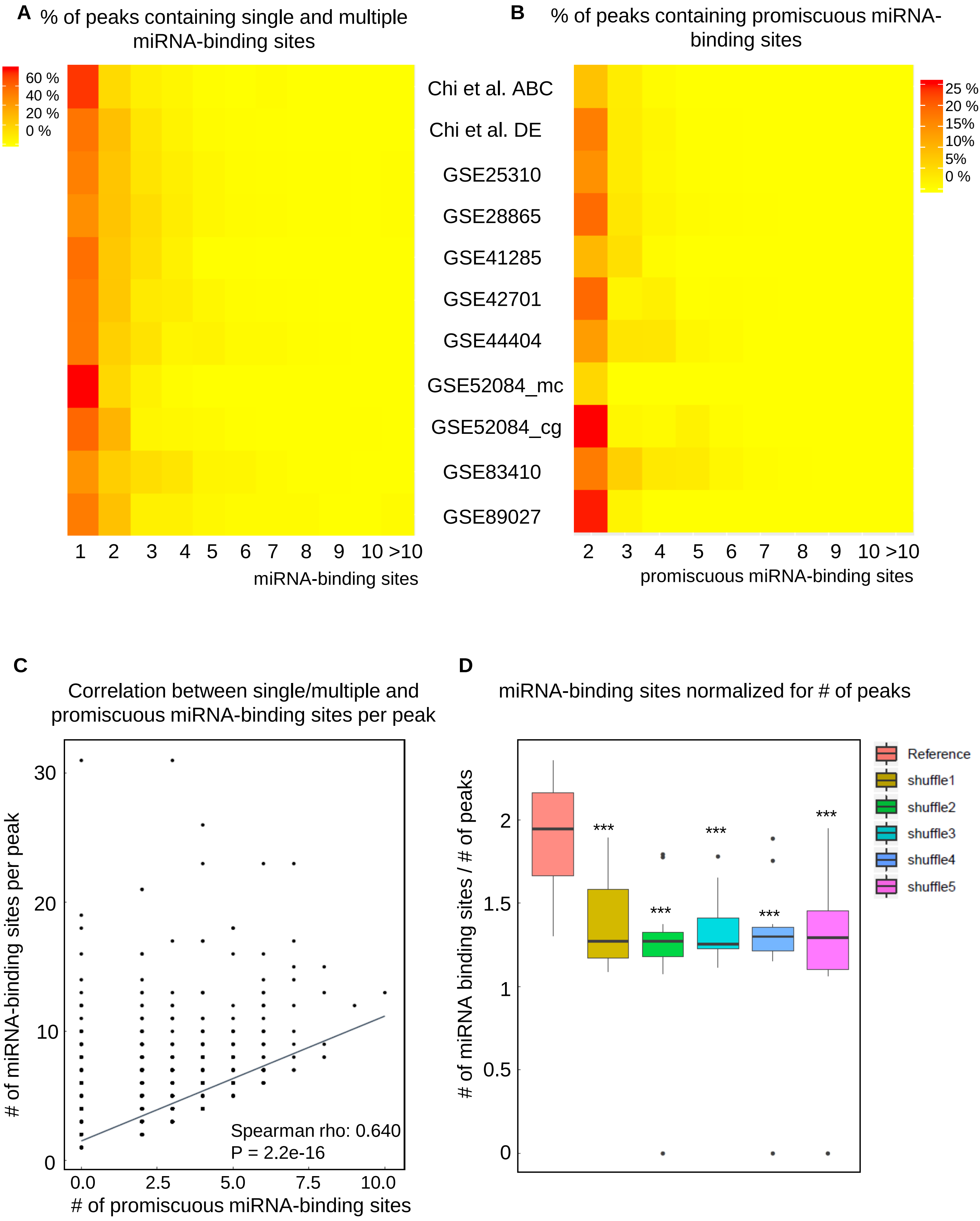

miRanda J20

Supplemental Figure 17

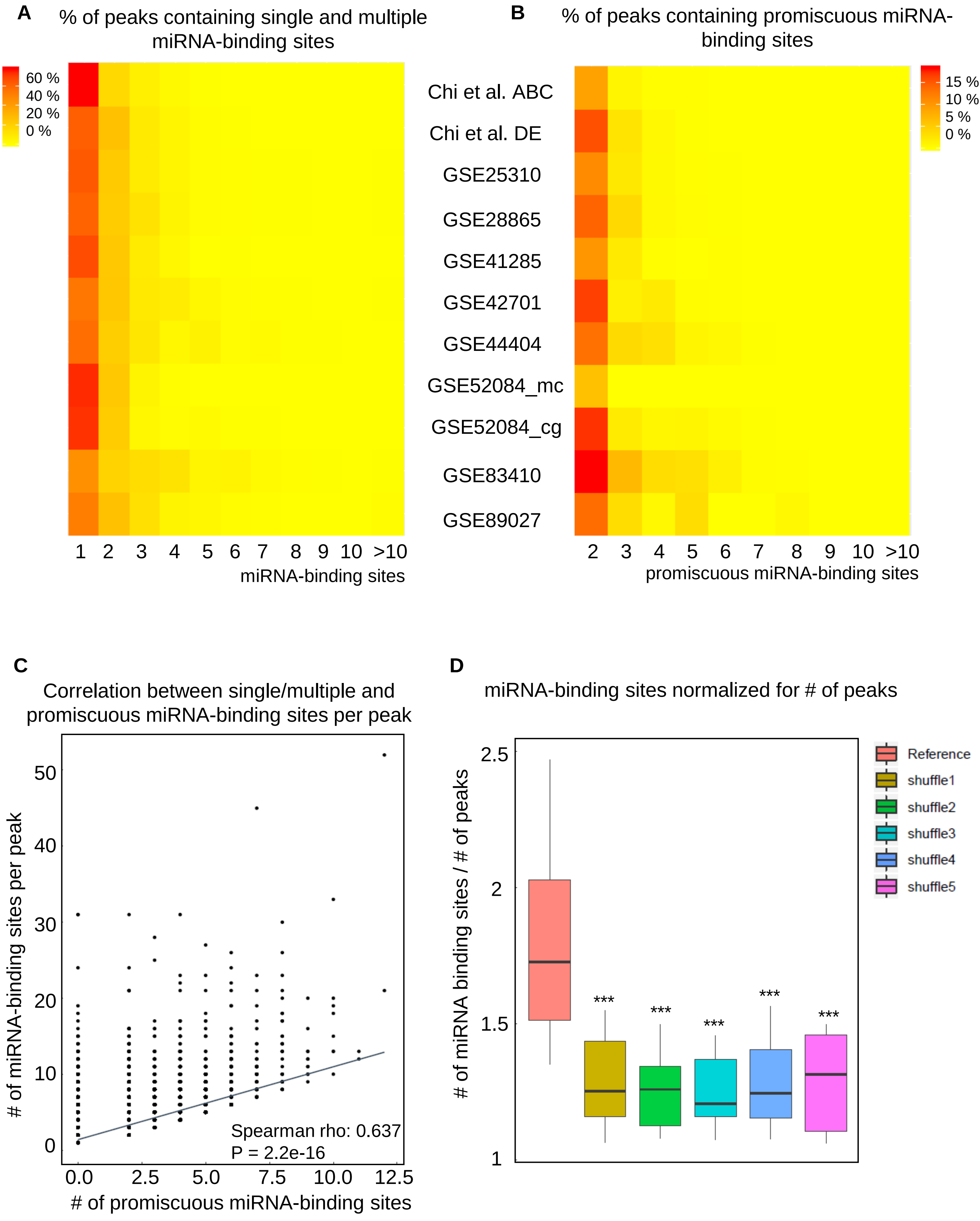

miRanda MERGE

Supplemental Figure 18

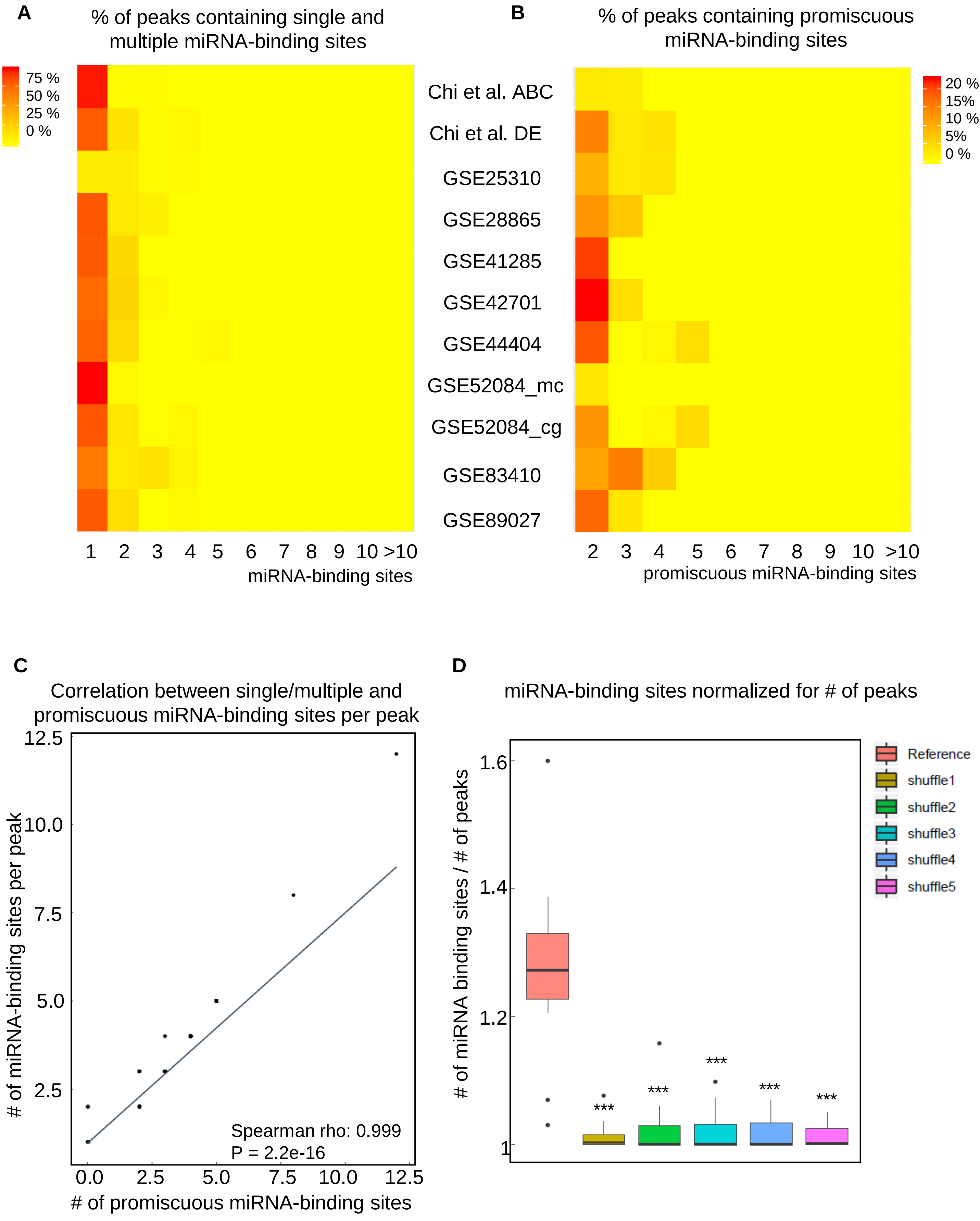

TargetScan J20

Supplemental Figure 19

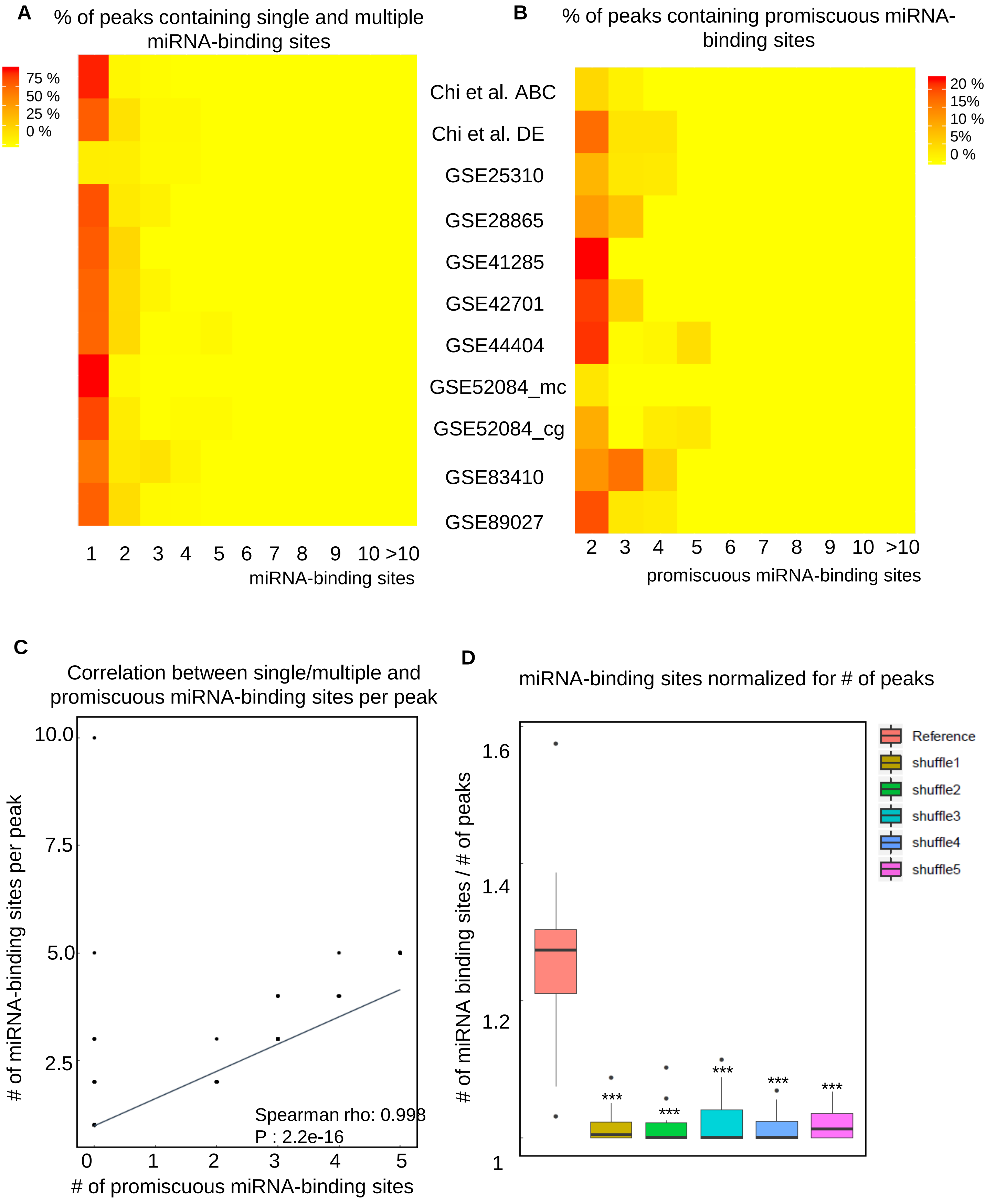

TargetScan MERGE

Supplemental Figure 20

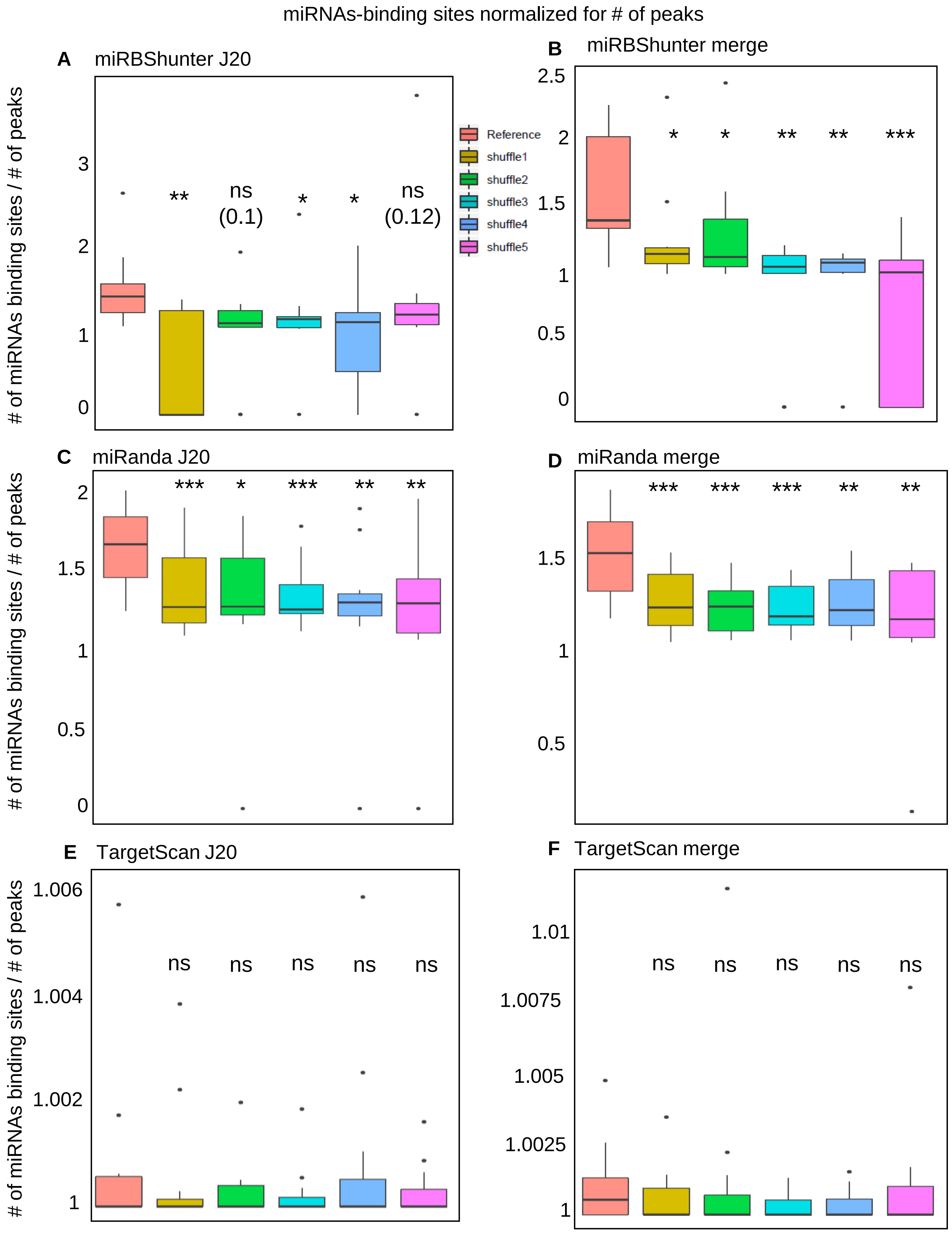

Supplemental Figure 21

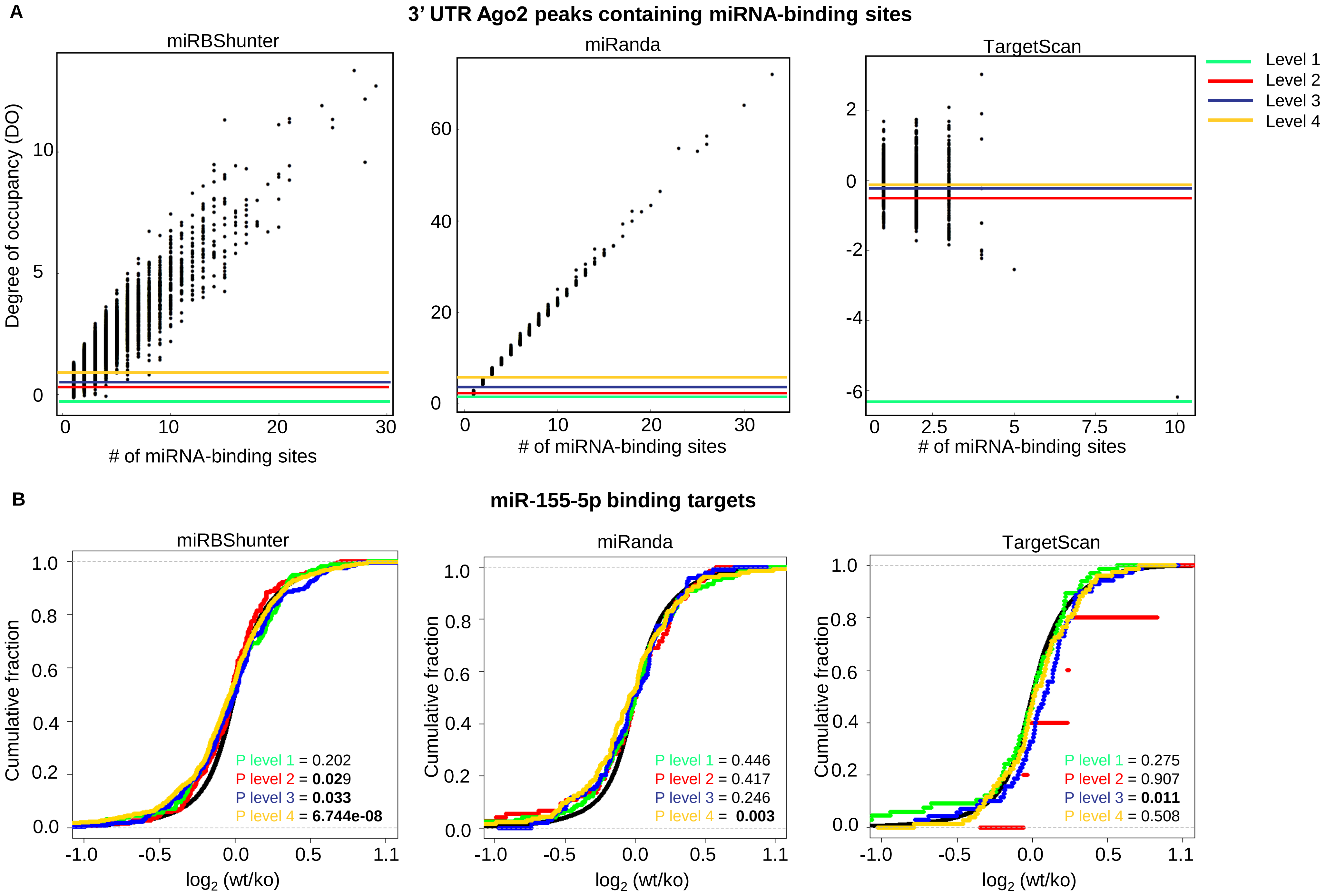

merge

Supplemental Figure 22

Distribution of distances between BS

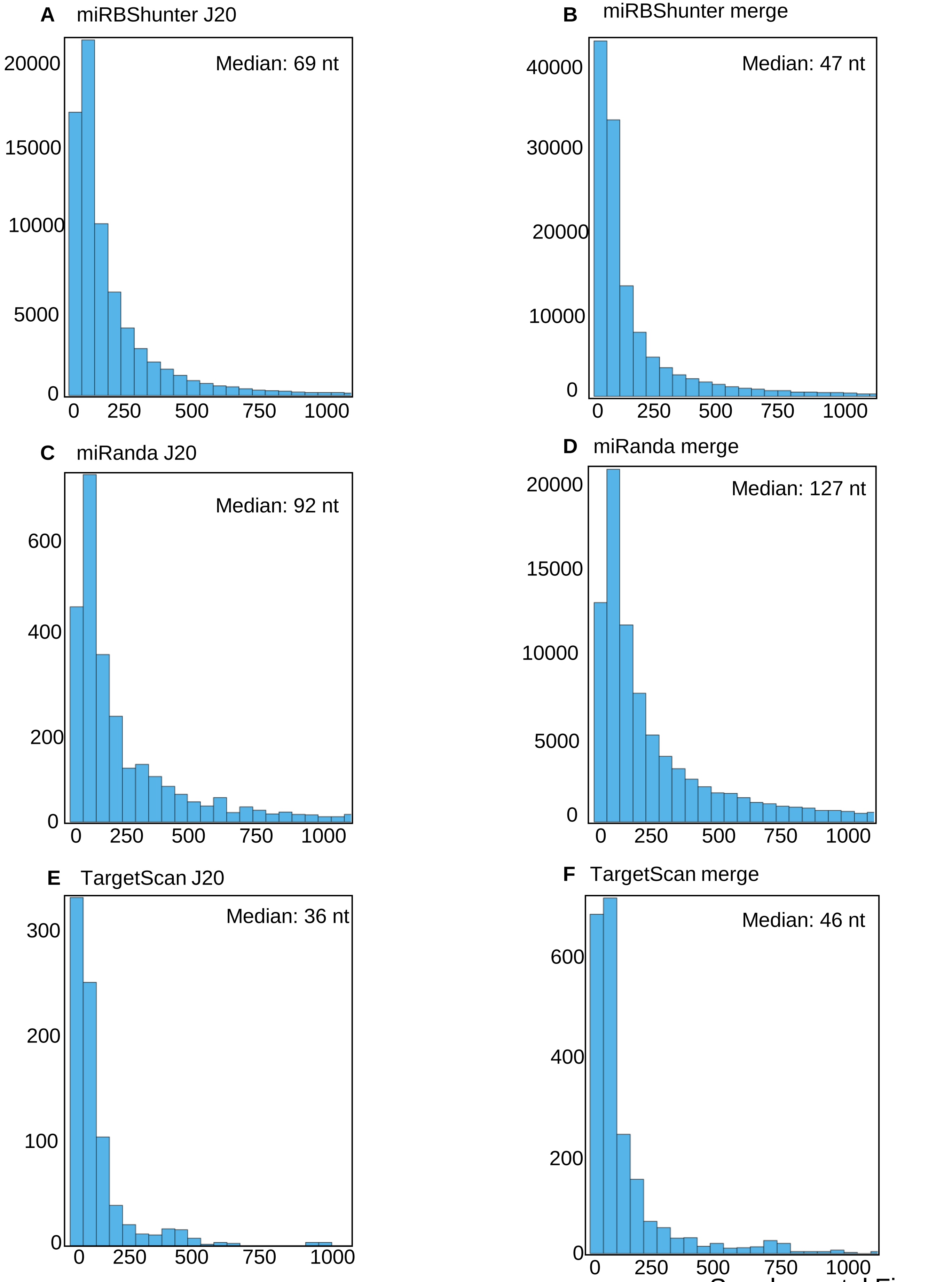

Supplemental Figure 23
