# Supplementary material for: Systemic CLIP-seq analysis and game theory approach to model microRNA mode of binding
Source: Nucleic Acids Res. 2021 Apr 6;49(11):e66. doi: 10.1093/nar/gkab198 (PMC8216473; doi:10.1093/nar/gkab198)
Supplement: gkab198_Supplemental_Files [file gkab198_supplemental_files.zip › Supplemental data.docx]

**Supplemental data**

**Supplemental data 1.**

Scrips for miGAME codes.

# R

#####calculate sum of marginal contribution (sum of DS)#########

library(dplyr)

data_sumDS <- aggregate(data$DS, by = list(peak = data$peakID, BS = data$BS_ID), FUN = sum)

colnames(data_sumDS)[3] <- "sumDS"

#####log transform of sum of DS######

data_sumDS [,c(3)] <- log10(data_sumDS [,c(3)])

colnames(data_sumDS)[3] <- "log_sumDS"

#####calculate sum of log transformed sum of DS######

data_DO <- aggregate(data_sumDS$log_sumDS, by = list(peak = data_sumDS$peak), FUN = sum)

colnames(data_DO)[2] <- "DO"

write.csv(data_DO, data_DO.csv")

**Supplemental data 2.**

Raw data about the Ago2 peak and miRNA-binding sites genomic locations can be downloaded from the following link.

<https://unice-my.sharepoint.com/:f:/g/personal/maria_stathopoulou_unice_fr/EociKJBjANhLjm2LEOvTYxYBBvd1ldr3FOiwXKQuYDl0tw?e=3s6BYd>
